# Reverse triggering ? a novel or previously missed phenomenon?

**DOI:** 10.1186/s13613-024-01303-4

**Published:** 2024-05-22

**Authors:** Robert Jackson, Audery Kim, Nikolay Moroz, L. Felipe Damiani, Domenico Luca Grieco, Thomas Piraino, Jan O. Friedrich, Alain Mercat, Irene Telias, Laurent J. Brochard

**Affiliations:** 1 Keenan Centre for Biomedical Research, Li Ka Shing Knowledge Institute and St. Michael’s Hospital, Unity Health Toronto, Toronto, ON Canada; 2 Interdepartmental Division of Critical Care Medicine, University of Toronto, Toronto, ON Canada; 3 Department of Respiratory Therapy, McGill University Health Centre, Montreal, QC Canada; 4 Departamento Ciencias de la Salud, Carrera de Kinesiología, Facultad de Medicina, Pontificia Universidad Católica de Chile, Santiago, Chile; 5 Department of Anesthesiology and Intensive Care Medicine, Catholic University of the Sacred Heart, Rome, Anesthesia Italy; 6 Emergency and Intensive Care Medicine, Fondazione Policlinico Universitario A. Gemelli IRCCS, Rome, Italy; 7 Department of Anesthesia, Division of Critical Care, McMaster University, Hamilton, ON Canada; 8 Medical ICU and Vent’Lab, University Hospital of Angers, University of Angers, 4 Rue Larrey, Angers Cedex 9, 49933 France

**Keywords:** Lung Protective Ventilation, Mechanical Ventilation, Reverse Triggering, Ventilator Asynchrony

## Abstract

**Background:**

Reverse triggering (RT) was described in 2013 as a form of patient-ventilator asynchrony, where patient’s respiratory effort follows mechanical insufflation. Diagnosis requires esophageal pressure (P_es_) or diaphragmatic electrical activity (EA_di_), but RT can also be diagnosed using standard ventilator waveforms.

**Hypothesis:**

We wondered (1) how frequently RT would be present but undetected in the figures from literature, especially before 2013; (2) whether it would be more prevalent in the era of small tidal volumes after 2000.

**Methods:**

We searched PubMed, EMBASE, and the Cochrane Central Register of Controlled Trials, from 1950 to 2017, with key words related to asynchrony to identify papers with figures including ventilator waveforms expected to display RT if present. Experts labelled waveforms. ‘Definite’ RT was identified when P_es_ or EA_di_ were in the tracing, and ‘possible’ RT when only flow and pressure waveforms were present. Expert assessment was compared to the author’s descriptions of waveforms.

**Results:**

We found 65 appropriate papers published from 1977 to now, containing 181 ventilator waveforms. 21 cases of ‘possible’ RT and 25 cases of ‘definite’ RT were identified by the experts. 18.8% of waveforms prior to 2013 had evidence of RT. Most cases were published after 2000 (1 before vs. 45 after, *p* = 0.03). 54% of RT cases were attributed to different phenomena. A few cases of identified RT were already described prior to 2013 using different terminology (earliest in 1997). While RT cases attributed to different phenomena decreased after 2013, 60% of ‘possible’ RT remained missed.

**Conclusion:**

RT has been present in the literature as early as 1997, but most cases were found after the introduction of low tidal volume ventilation in 2000. Following 2013, the number of undetected cases decreased, but RT are still commonly missed.

**Prior Abstract:**

Reverse Triggering, A Missed Phenomenon in the Literature. Critical Care Canada Forum 2019 Abstracts. *Can J Anesth/J Can Anesth* **67** (Suppl 1), 1–162 (2020). https://doi-org.myaccess.library.utoronto.ca/10.1007/s12630-019-01552-z.

**Supplementary Information:**

The online version contains supplementary material available at 10.1186/s13613-024-01303-4.

## Introduction

Patient ventilator asynchronies are common and have been associated with longer duration of mechanical ventilation [1, 2] and higher mortality [3]. The impact and prevalence of asynchrony is often under-estimated given challenges in identification [4].

Reverse triggering (RT) is a form of asynchrony described by Akoumanaki et al. in 2013, occurring in up to 30–55% of sedated patients on controlled or assist-control ventilation [5]. RT is defined as a patient inspiratory effort occurring after the onset of mechanical insufflation, appearing to be triggered by the ventilator’s insufflation [6]. While the mechanisms leading to RT are not fully understood, a frequent mechanism is that of respiratory entrainment; the establishment of a fixed, repetitive rhythm between a patients respiratory pattern generator and an external stimulus (here mechanical insufflation) [5]. The physiological consequences of RT are not yet known. In an animal model, only when the respiratory effort was large, RT was associated with diaphragm dysfunction [7]. RT can increase tidal volumes in pressure-regulated modes of ventilation [8]. The reverse triggered patient effort may be significant enough to trigger a second breath before complete exhalation, also known as breath stacking [9]. This appears similar to double triggering, where a patients effort triggers the first ventilator breath and is sustained long enough to trigger a second breath after the ventilator cycles off. Both reverse triggering and double triggering can lead to injurious ventilation due to breath stacking, albeit via distinct underlying mechanisms [10]. Therefore, RT can be present without (most frequently) or with breath-stacking. The circumstances facilitating RT are not fully understood but have been associated with sedation [10] and low tidal volume ventilation [5, 11, 12]. In experimental models RT could be easily induced using reduced tidal volume [7].

**Fig. 1:**
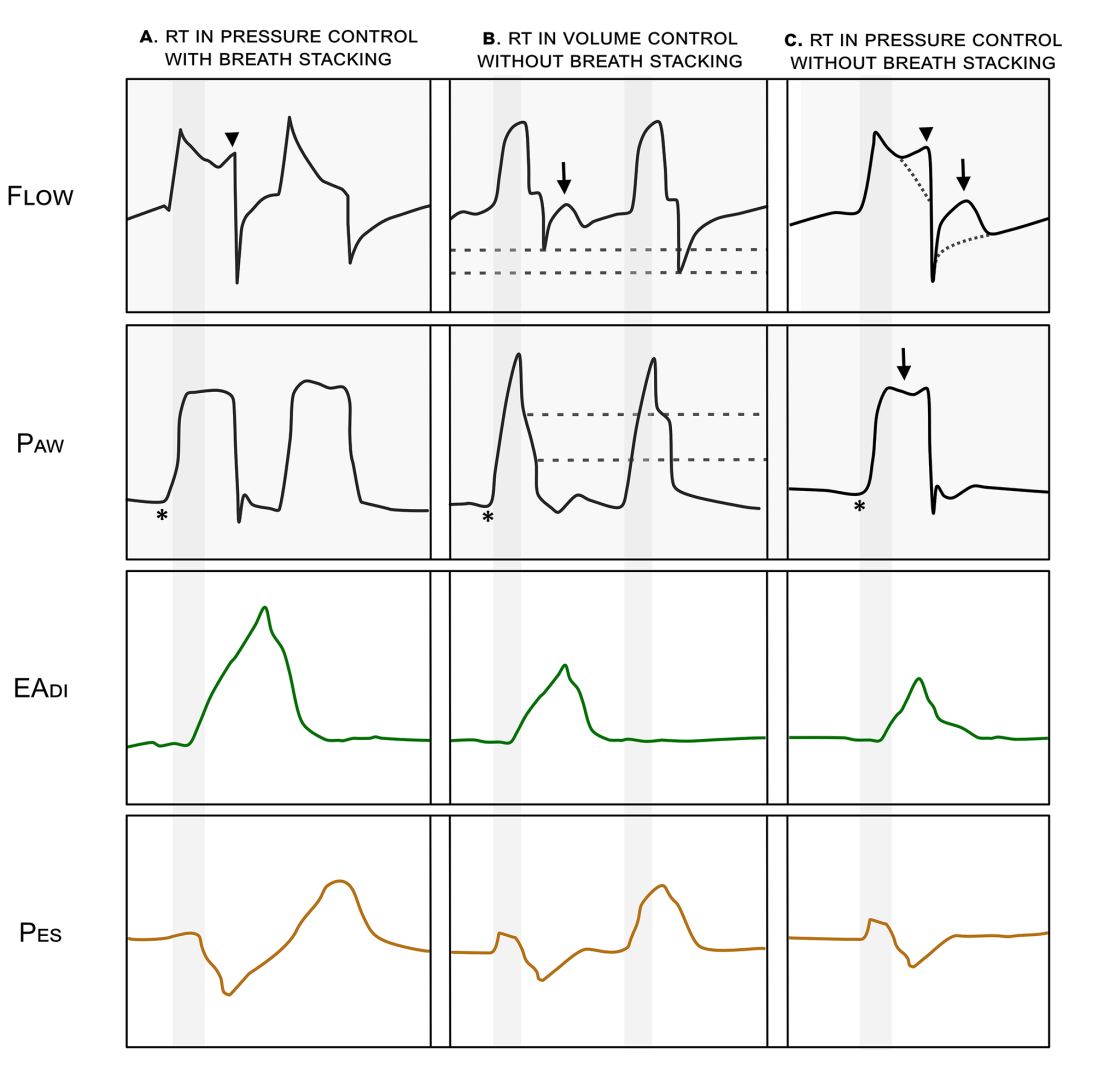
Example tracings of waveforms demonstrating diagnostic features of reverse triggering (RT). RT can be diagnosed using only the ventilator waveform in either volume or pressure-controlled modes. The absence of negative deflection in the airway pressure waveform (*) indicates that these are mandatory breaths. **A** demonstrates breath stacking after a mandatory breath in pressure control mode. The stacked breath following a passive mechanical insufflation due to a mandatory breath must be initiated by a patient effort, therefore this finding is consistent with RT. Additionally, the increase in flow towards the end of the breath (arrowhead) indicates patient effort). **B** shows a mandatory volume-controlled breath with reverse triggering, followed by a mandatory breath without RT. There is a sudden decrease in expiratory flow (black arrow) a decrease in peak expiratory flow (dashed line, flow waveform) and plateau pressure (dashed line, pressure waveform), all corresponding to a patient effort. **C** demonstrates a mandatory breath with reverse triggering in pressure control mode. The dotted line represents the anticipated morphology of a normal mandatory breath. There is an increase in end-inspiratory flow (arrowhead), a decrease in expiratory flow (arrow – flow waveform) and a subtle drop in peak pressure (arrow – pressure waveform) all representing patient effort. The addition of esophageal pressure monitoring (*Pes*) provides a precise indicator of the delay between onset of mechanical insufflation and the beginning of patient effort (*vertical grey column).* This is used to diagnose ‘**Definite RT’.**

The gold standard for diagnosis uses a direct measure of patient respiratory muscle activity, e.g., diaphragmatic electrical activity (EA_di_) or esophageal pressure (P_es_). RT is diagnosed when the patient’s respiratory effort is initiated after the start of mechanical insufflation. Frequently, this may occur in a regular, recurring pattern, suggesting that the patient’s respiratory cycle is ‘entrained’ to the set rate of the ventilator, but this may not always be the case and the pattern can appear irregular [5]. In the absence of such monitoring, RT can still be detected in the flow and pressure ventilator waveform characteristic of RT [13]. (Fig. 1); these changes are sometimes subtle and may be easily missed.

While RT was only recent described in critically ill patients [6], it is likely that this phenomenon was already present and often missed. There is no large historical database of recordings that would allow measurement of the real burden of this phenomenon retrospectively, however there are publications including respiratory waveforms from which it may be possible to estimate the historical burden of this phenomenon. We wondered whether (1) RT in earlier publications may have gone undetected, with or without attribution to another phenomenon, (2) RT may still be undetected and/or attributed to another type of asynchrony in the absence of direct monitoring of the onset of patient effort. (3) the novelty of this phenomenon may also be related to the era of lung protective ventilation which was generalized mostly after 2000 [14]. In this study we conducted a systematic review of the literature to identify the prevalence of undetected RT (either attributed to other phenomena or not) in previously published figures with ventilator waveforms. We analyzed cases prior to and after the initial description of RT in 2013, as well as before and after the introduction of low tidal volume ventilation in 2000, to examine any temporal relationship related to this change in ventilation strategy. Finally, we describe cases where RT was attributed to different phenomenon to better understand the nature of misattribution.

## Methods

### Eligibility criteria

We started from all previously published articles potentially describing ventilator waveforms in detail and where RT was expected to be possibly present and described if present, i.e., those including patients on mechanical ventilation and discussing issues related to patient-ventilator asynchrony (see search strategy, supplementary Fig. 1). After abstract screening, when full texts were retrieved, we included all studies and supplementary material that contained a ventilator waveform (with at least flow and airway pressure waveforms present, with or without Pes or EAdi) from a patient on any mode of controlled or assist-control ventilation, with a minimum recording of two consecutive breaths. The terms controlled ventilation and assist-control ventilation are interchangeably used in the literature, but we were looking for mandatory pre-determined breaths triggered or not by the patient. Manuscripts were excluded if studies involved only animal, neonate or pediatric patient population, negative pressure ventilation, high frequency oscillation or spontaneous modes of ventilation (i.e., partial assistance like pressure support ventilation).

### Database search

We searched Medline, EMBASE and the Cochrane Central Register of Controlled Trials for eligible articles between 1950 and 2017, using keywords related to asynchrony. Our search strategy is listed in the supplement (supplementary Fig. 1). Four authors (IT, LFD, DLG, TP) independently screened eligible abstracts using Covidence® systematic review tool.

### Data collection process

Articles were then scanned for eligible figures. Figures were analyzed independently by three authors (RJ, AK, NM). Figures from eligible manuscripts were excluded if there was insufficient information on the figure (single breath recording, isolated waveform of flow or airway pressure), waveforms from animals, test lungs, simulated patients, neonates or pediatric patients, schematic representations, or from spontaneous modes of ventilation (i.e., pressure support, neurally adjusted ventilatory assist, proportional assist ventilation, synchronised intermittent mandatory ventilation). Following application of exclusion criteria, all included figures contained a waveform from a human patient ventilated in assist-control or controlled mode of ventilation. These will be subsequently referred to as “waveforms” for consistency.

Waveforms from assist-control mechanical ventilation modes were classified as those with spontaneous effort (when the breath is triggered by patient effort) or mandatory breaths (where there was no patient effort and the machine triggers the breath). All figure legends were reviewed for mention of reprint and duplicate waveforms were excluded.

### Waveform analysis

Each waveform constituted our unit of analysis. Mandatory breath tracings were categorized into those with no identified RT, with *‘possible’ RT* (Paw and Flow waveforms demonstrating changes consistent with RT), or with *‘definite’ RT* (Pes or Eadi showing a patient inspiratory effort starting after mechanical ventilation) (Fig. 1).

*RT* was defined as evidence of onset of patient effort beginning after the initiation of a mandatory breath by the ventilator. The patient effort in RT should be temporally related to the onset of mechanical insufflation. Given that not all figures included a timescale, it was not possible to use an absolute cut off. Instead, if the patient effort was visually within the first 50% of the whole respiratory cycle, it was considered to be RT, as opposed to ineffective effort.

*‘Definite RT’* was used where a method such as esophageal pressure or electrical activity of the diaphragm monitoring were used to directly demonstrate patient effort occurring after onset of mechanical insufflation.

*‘Possible RT’* was used where a patient effort beginning after the onset of mechanical insufflation was evident from changes in the flow and pressure waveforms from the mechanical ventilator. The criteria to diagnose ‘*possible RT’* were as follows:

1. Evidence of a mandatory breath delivered by the ventilator. Determined by:
  - The absence of a negative deflection in the pressure-time curve immediately preceding the breaths.
  - Indication in the text or figure legend as to whether breaths were mandatory, or patient initiated.

#### AND at least one of

(2) Breath stacking following the mandatory breath.
(3) Changes in the expiratory flow waveform suggesting patient effort.
  - A reduction in peak expiratory flow compared to other breaths.
  - A positive deflection in expiratory flow disrupting the normal exponential decay of expiration.
(4) Changes in the inspiratory phase.
  - during volume-control ventilation.
    i. a negative deflection in airway pressure.
  - during pressure control ventilation.
    i. a negative deflection in airway pressure.
    ii. a positive deflection in inspiratory flow on the flow-time curve.

Expert assessment was compared to the author’s descriptions of the waveforms either in the figure legends or the text. Waveforms with evidence of RT were labelled as *‘undetected’* if the figure legend or text did not describe RT or attributed the event to a different phenomenon. Disagreement was resolved by consensus, and all waveforms with RT were double-checked by the same authors (IT, LB).

### Additional data extraction

Clinical data were obtained for cases of RT where available, either from figure legends or the manuscript where applicable.

### Outcomes

Our primary outcome was the incidence of undetected cases of RT in the previously published literature, before and after the date of initial description in 2013. Secondary outcomes included (a) the proportion of undetected cases in the subgroups of ‘possible’ and ‘definite’ RT, (b) a summary of the terms used to describe undetected cases in the figure legends, and (c) comparison of prevalence of ‘possible’ and ‘definite’ RT before and after 2000.

### Statistical analysis

Analysis of the extracted waveforms was descriptive, including total number of identified cases of RT, and proportions of cases that were ‘definite’ versus ‘possible’. Categorical variables were analyzed using a chi squared (X2) test. Data analysis and creation of figures was done using Microsoft Excel (Version 2311).

## Results

### Study selection

**Fig. 2:**
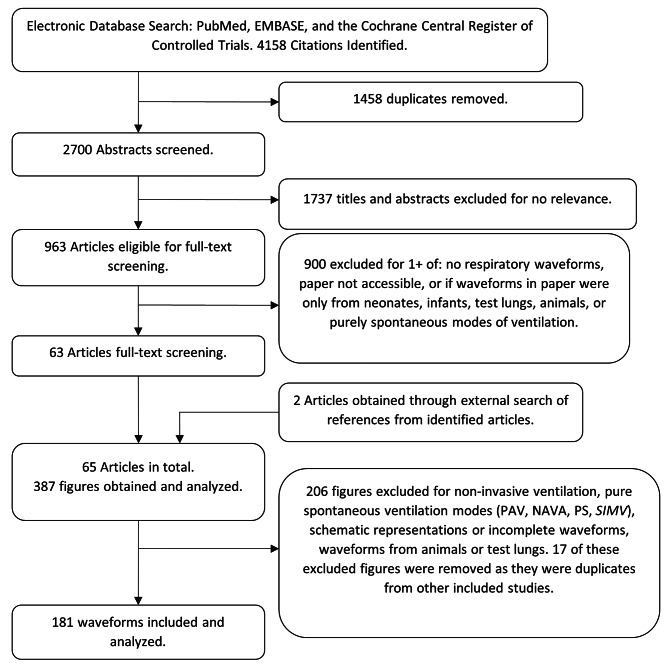
Search Strategy. PAV = Proportional Assist Ventilation. NAVA = Neurally Adjusted Ventilatory Assist. PS = Pressure Support. SIMV = Synchronized Intermittent Mandatory Ventilation

The search extracted 2700 articles and the selection process is described in Fig. 2. After applying our selection criteria, we identified 63 eligible articles for full review, and 2 articles were subsequently identified through an external search that comprised review of references from studies identified in the initial search. A total of 387 figures were assessed, with 206 figures excluded. Of these excluded figures, 17 were excluded as duplicates from other included articles. In total, 181 eligible waveforms were identified for analysis.

### Study/Waveform characteristics

**Fig. 3:**
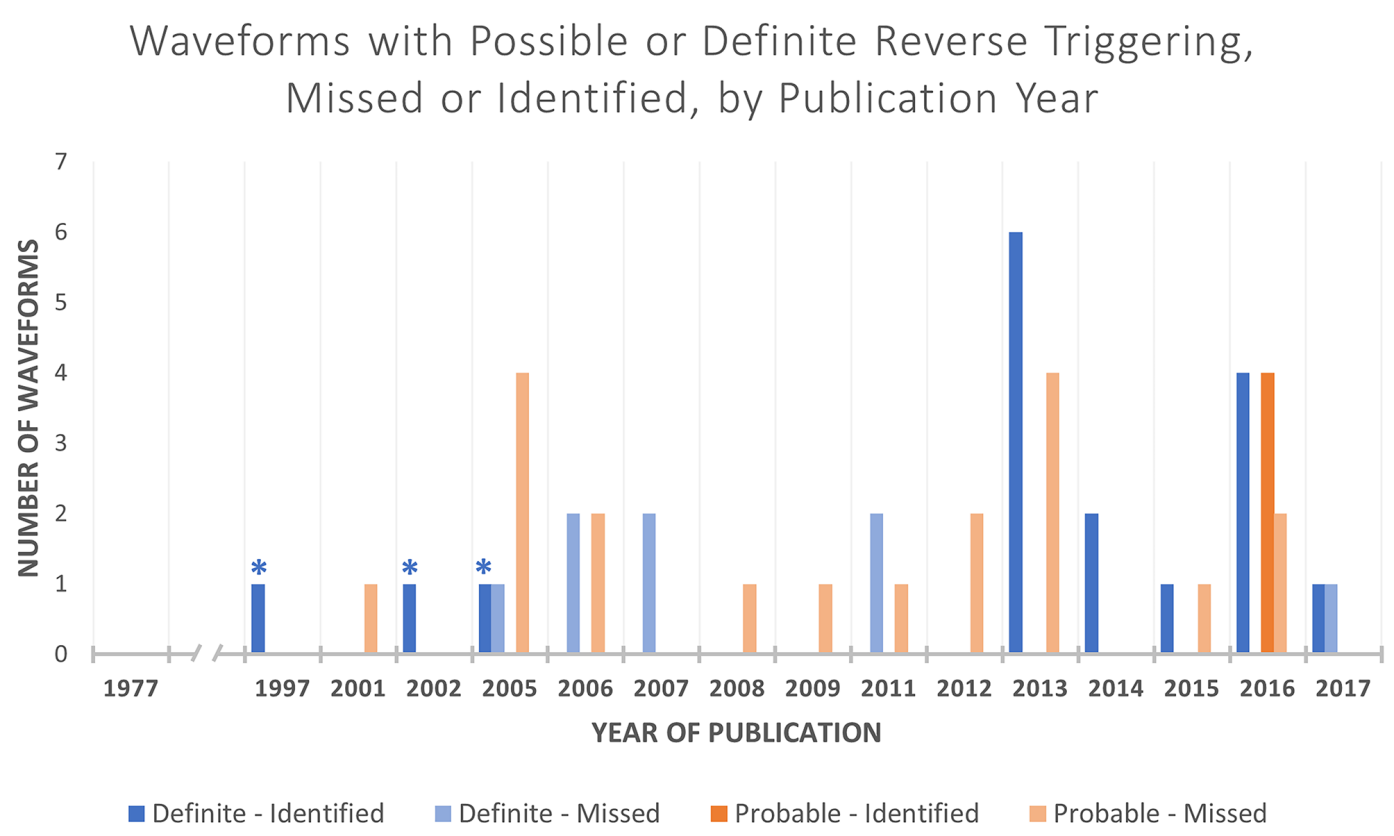
Reverse triggering cases by year published * = Cases published prior to 2013 that correctly describe reverse triggering without specifically naming it

In total, 65 eligible articles were included in this study. The publication dates ranged from 1977 to 2017 (Fig. 3). These included literature reviews, case reviews/series, prospective clinical studies, prospective observational studies, diagnostic testing studies and a prospective randomised clinical trial. Table 1 describes the study characteristics and breakdown of included waveforms [1, 4, 6, 15–78]. Where available, clinical details for cases of RT can be found in Table 2.

**Table 1:** A summary of details of papers from literature search included in the final analysis

Author	Year	Journal	Type of Article	Total waveforms included / Total Figures in Paper
Harboe [15]	1977	Acta Anaesthesiologica Scandinavica	Review	3/3
Popova [16]	1980	Resuscitation	Case series	4/4
Siegel [17]	1985	Annals of Surgery	Prospective clinical study	2/15
Tokioka [18]	1989	Intensive Care Medicine	Prospective clinical study	2/2
Blackson [20]	1995	Respiratory Care	Case study	2/4
Branson [21]	1996	Respiratory Care	Review	2/10
Dick [22]	1996	Clinics in Chest Medicine	Review	2/4
Chao [23]	1997	Chest	Prospective Cohort	3/3
Leung [24]	1997	American Journal of Respiratory and Critical Care Medicine	Prospective clinical study	2/7
Tobin [25]	1998	Molecular and Cellular Biochemistry	Review	1/12
Imanaka [26]	2000	Critical Care Medicine	Prospective Clinical Study	1/2
Babuska [27]	2001	Artificial Intelligence in Medicine	Prospective Cohort, Physiologic Study	1/6
Officer [28]	2001	Chest	Case study	1/1
Sassoon [29]	2001	Current Opinion in Critical Care	Review	2/8
Fontes [30]	2002	Current Opinion in Anaesthesiology	Review	1/4
Kallet [31]	2002	Respiratory Care	Case study	1/1
Younes [32]	2002	American Journal of Respiratory Critical Care Medicine	Prospective clinical study	3/10
Kondili [33]	2003	British Journal of Anaesthesia	Review	5/17
Blanch [34]	2005	Respiratory Care	Review	4/17
Bonetto [35]	2005	Respiratory Care Clinics of North America	Review	1/3
Dhand [36]	2005	Respiratory Care	Review	1/16
Kallet [37]	2005	Respiratory Care	Prospective Clinical Study	4/7
Nilsestuen [38]	2005	Respiratory Care	Review	11/54
Georgopoulos [39]	2006	Intensive Care Medicine	Review	6/61
Kallet [40]	2006	Critical Care Medicine	Prospective Clinical Study	1/2
Thille [1]	2006	Intensive Care Medicine	Prospective cohort	1/4
Chen [41]	2007	Intensive Care Medicine	Case Study / Correspondence	1/1
Thille [42]	2007	Clinical Pulmonary Medicine	Review	6/7
Thille [43]	2007	Réanimation	Review	3/3
Thille [44]	2007	Intensive Care Medicine	Case / correspondence	1/1
Chen [45]	2008	Critical Care Medicine	Prospective Clinical Study	2/3
Pohlman [46]	2008	Critical Care Medicine	Prospective cohort	2/4
De Wit [47]	2009	Critical Care Medicine	Prospective Cohort	2/4
De Wit [48]	2009	Journal of Critical Care	Prospective cohort	4/5
Kondili [49]	2009	Expert Review of Respiratory Medicine	Review	1/19
Mellot [50]	2009	Critical Care Nurse	Review	3/4
Unroe [51]	2010	Current opinion in Critical Care	Review	1/4
Colombo [52]	2011	Critical Care Medicine	Prospective Observational	3/4
De Wit [53]	2011	Respiratory Care	Review	3/10
Liao [54]	2011	Respiratory Care	Prospective cohort	3/4
Blanch [55]	2012	Intensive Care Medicine	Diagnostic Test Study	3/8
Chacón [56]	2012	American Journal of Critical Care	Diagnostic Test Study	1/1
Correger [57]	2012	Medicina Intensiva	Review	4/24
Laghi [58]	2012	Minerva Anesthesiologica	Review	2/14
Akoumianaki [6]	2013	Chest	Case Series	6/9
Branson [59]	2013	Respiratory Care	Review	4/6
Chanques [60]	2013	Critical Care Medicine	Prospective Cohort, Physiological Study	3/7
Murias [61]	2013	Minerva Anaesthesiologica		2/7
Richard [62]	2013	Intensive Care Medicine	Bench and prospective clinical study	1/4
Akoumianaki [63]	2014	American Journal of Respiratory and Critical Care Medicine	Review	3/6
Brochard [64]	2014	Current Opinion in Critical Care	Review	1/2
Mellot [65]	2014	Heart & Lung	Prospective Observational	12/24
Chiew [66]	2015	IEEE	Prospective Clinical Study	1/3
Mietto [67]	2015	Anaesthesiology Intensive Therapy	Review	3/5
Yonis [68]	2015	Intensive Care Medicine	Case report	1/1
Delisle [69]	2016	American Journal of Respiratory and Critical Care Medicine	Case series / correspondence	2/2
Dres [4]	2016	Current Opinion in Critical Care	Review	2/3
Figueroa-Casas [70]	2016	Annals of the American Thoracic Society	Prospective clinical study	6/9
Mauri [71]	2016	Intensive Care Medicine	Review	6/10
Murias [72]	2016	Intensive Care Medicine	Review	4/4
Restrepo [73]	2016	Clinics in Chest Medicine	Review	2/22
Guervilly [74]	2017	Intensive Care Medicine	Prospective randomised controlled study	2/5
Mechati [76]	2017	Annals of Intensive Care	Prospective observational	2/2
Sangha [77]	2017	Journal of Intensive Care Medicine	Case Series	1/7
Tripathi [78]	2017	A & A: Case Reports	Case Report	5/5

**Table 2:** A summary of clinical details related to waveforms that demonstrate reverse triggering

Author	Type of Study	Patients with RT (*N*)	Age, Years (Range)	Pathologies	Ventilator Mode (*N*)	PEEP (mean)	RR (mean)	Tidal Volume (mean)	Sedation Score	Pplat (or Pinsp)	PF ratio
Kallet, 2002	Case Discussion	1	55	ARDS - Pneumonia, Septic Shock	VAC	16	32	5 mL/kg		30	77
Kallet, 2005	Prospective non-randomized trial	2	39–72	ARDS (Pneumonia, Sepsis), ALI- Polytrauma	VC (1), PCV (1)	9.5		6 mL/kg			150 (90–210)
Kallet, 2006	Prospective non-randomized trial	1	41	ARDS - Sepsis	VC	5		6 mL/kg	Ramsey 4		
Chen, 2007	Case Report	1		Heart failure and pneumonia	PCV		25				
Liao, 2011	Prospective cohort	12	43–89	Sepsis (3), Pneumonia (4), COPD (3), VAP (1), CHF (1)	VC (10), PC (2)			8.2 mL/kg	Ramsey 2 (1), Ramsey 3 (2). Ramsey 4 (1). Ramsey 5 (3), Coma (3)		303
Akoumianaki, 2013	Retrospective Cohort	8	25–73	ARDS (Pneumonia, Sepsis, SIRS, Polytrauma)	PAC (3), VAC (5)	10.7	22	6.8 mL/kg	RAS − 4 to -5	26.1	161.9
Chanques, 2013	Physiological study	2	66	COPD	VC			400 mL			
Yonis, 2015	Case Report	1	50	ARDS - Sepsis	VC	13	24	340 mL	Ramsey 6	23	117
Delisle, 2016	Case study	2	40–78	Out of hospital cardiac arrest and Brain Death	VAC (2)	5	15	480 mL			
Murias, 2016 (ICM)	Review article	4		acute respiratory failure	VC (2), PC (2)	6.75	17.5	397.75 mL	RAS 1 (3). RAS 4 (1)	19.5	
Guervilly, 2017	Prospective RCT	1		ARDS	VC			6 mL/kg			
Mechati, 2017	Abstract - blinded RCT	1		ARDS							

There were 4 manuscripts with incomplete clinical details, including 5 waveforms demonstrating RT, all of which were seen during VCV. There were 17 manuscripts in which 20 figures demonstrated RT, but no relevant clinical data was described. Where blank, data was not reported

RT = Reverse Triggering, PEEP = Positive end expiratory pressure, RR = Set Respiratory Rate, Pplat = Plateua pressure, Pinsp = Peak Inspiratory Pressure, PF ratio = ratio of partial pressure of oxygen in arterial blood (PaO2) to the fraction of inspiratory oxygen concentration

### Waveform analyses and undetected RTs

**Fig. 4:**
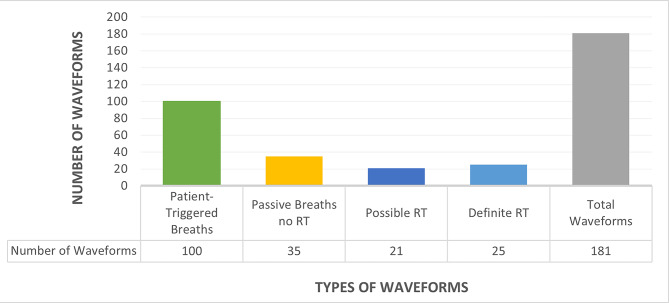
Breakdown of the categorization of included waveforms

We retrieved 181 eligible waveforms published from 1977 to 2017 with 23 waveforms (12.7%) published prior to the year 2000 (Fig. 3). 46 waveforms (25.4%) showed evidence of RT, 45 being after 2000 (prevalence of RT 4% vs. 28%, X^2^ = 4.6, *p* = 0.03). We classified 21 of these 46 as ‘possible’ RT and 25 as ‘definite’ RT (Fig. 4) Overall, there were 25 of the 46 waveforms with evidence of RT (54%) that were undetected. 17 out of 21 cases of ‘possible’ RT were undetected (80.1%) and 8 out of 25 cases of ‘definite’ RT were undetected (32%).

The figure legends for undetected cases of RT were extracted and collated in Table 3. ‘Double Triggering’ was the term most often mentioned in the description of the cases. The term breath-stacking was also often used, but in association with a mechanism other than reverse triggering.

**Table 3:** Figure legends from pre-2013 that identified RT based on an accurate description of the phenomenon

Article	Pre-2013 – Identified Reverse Triggering
**Leung et al., 1997**	‘The final breath was machine-initiated, and patient effort commenced after the onset of flow delivery’
**Kallet et al., 2002**	‘Her inspiratory efforts appeared to commence at peak inspiration, and intermittently she was able to trigger extra mechanical breaths’
**Kallet et al., 2005**	‘These waveforms reflect a common observation during lung-protective ventilation, whereby a ventilator-triggered breath stimulated the patient’s spontaneous breathing effort.’

### 2013 timepoint and definitive versus possible RTs

**Fig. 5:**
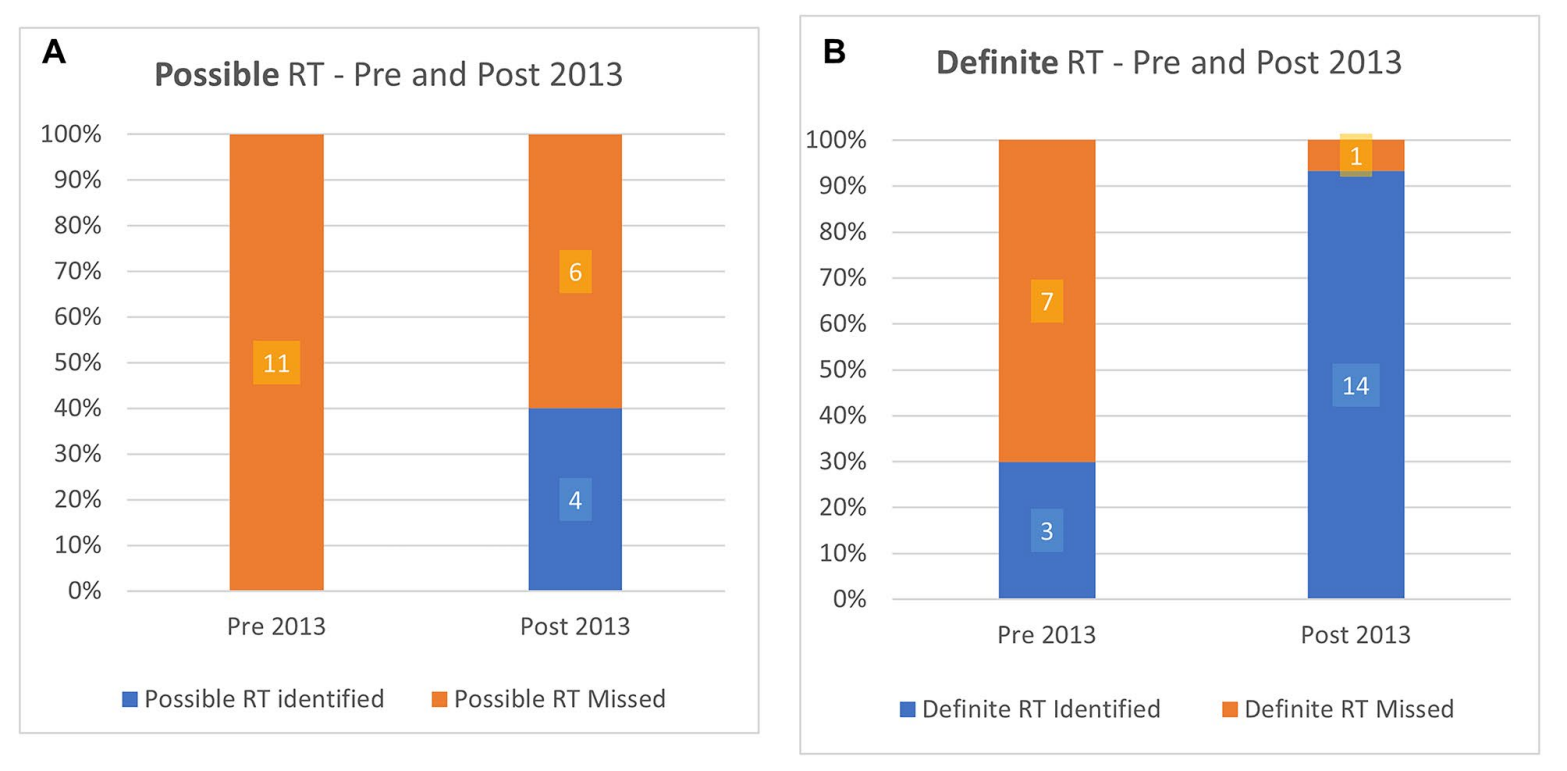
Reverse triggering (RT) cases detected by authors, by time period (pre and post initial description in 2013). **(A)** Cases of ‘Possible’ RT (i.e. without the use of Pes or EAdi) categorized as detected (by authors) or un-detected. Pre-2013, all cases of possible reverse triggering were misattributed. Post-2013 a significant proportion continued to be misattributed. **(B)** Cases of ‘Definite’ RT (i.e. using (Pes or Eadi), demonstrating that pre-2013 a select few cases correctly described the phenomenon before it’s definite description in 2013. Following 2013, most cases are detected when using definitive monitoring. *Pes: Esophageal pressure, EAdi: Electrical activity of the diaphragm*

112 out of 181 waveforms were published pre-2013. In total, 21 out of 112 waveforms from 1997 to 2012 had evidence of RT (18.7%), with 11 ‘possible’ RT and 10 ‘definite’ RT. Most RT cases were not defined as RT (18 undetected RT cases pre-2013, 85.7% of total waveforms, Fig. 5). Three out of 10 ‘definite’ RT cases [24, 31, 37] described the pattern and findings of RT in their respective figure legends without specifically naming it (as the phenomenon had not yet been named as RT) (Table 4). Of these cases, the description by Kallet et al. in 2005 [37] is closest to the current definition of RT: “These waveforms reflect a common observation during lung-protective ventilation, whereby a ventilator-triggered breath stimulated the patient’s spontaneous breathing effort”.

**Table 4:** Terms used in waveform descriptions in undetected ‘possible’ and ‘definite’ cases of reverse triggering

**‘POSSIBLE’ RT**	**Number of Mentions**
**Double Triggering**	6
**Breath Stacking**	3
**Inadequate Ventilator Flow**	2
**Ineffective Efforts**	1
**Premature Cycling**	2
**Trigger Dyssynchrony**	1
**Double Cycling**	1
**Other/No Mention**	2
**‘DEFINITE’ RT**	**Number of Mentions**
**Double Trigger**	4
**Ineffective Efforts**	2
**Pseudo-double triggering**	1
**Auto-triggering**	1
**Other/No Mention**	1

Post 2013, there were 10 cases of ‘possible’ RT, of which 6 were undetected. 15 cases of ‘definite’ RT were found and only 1 was undetected (Fig. 5). The total number of undetected cases post-2013 was 7 (28% of total waveforms with identified RT in this period). Undetected cases dropped dramatically (Fig. 4).

## Discussion

In this systematic review of ventilator waveforms included in manuscripts related to ventilator asynchrony, RT was found to be present frequently in patients identified by the authors for having asynchrony on control or assist-control ventilation. A significant proportion of cases of RT were undetected or attributed to other phenomena. The recognition increased substantially after the 2013 publication, but especially when an additional physiological signal was available. The diagnosis remains challenging when using only the ventilator waveforms. The prevalence of RT has been much higher after 2000 in the low tidal volume era.

As expected, undetected cases were published prior to the first time the phenomenon was named RT in 2013 even in the presence of definitive monitoring such as Pes or EAdi. Interestingly the phenomenon was already described long before that publication without gaining much attention [24, 31, 37]. In contrast, post-2013, almost all published waveforms that had definitive monitoring correctly identified the phenomenon and named it RT. In the absence of definitive monitoring, there continued to be a significant proportion of ‘possible’ RT that was undetected post-2013. Given the subtle changes in ventilator waveforms that differentiate RT, it is not surprising that it remains difficult to diagnose at the bedside in absence of definitive techniques. This suggests the potential benefit of monitoring esophageal pressure and/or EAdi in the diagnosis and management of asynchronies or the need for automated methods [13]. Often reverse triggering was mistaken for double triggering and “breath stacking”. There is no direct evidence that monitoring techniques can reduce asynchrony during controlled or assist-control modes of ventilation, but a direct monitor could theoretically improve differentiation between these distinct phenomena, which is the first step towards effective management.

While we are not able to estimate the true prevalence of RT based on this study, it is of clinical interest that the rate of undetected RT remains relatively high even amongst the relatively recently published waveforms related to ventilator asynchrony. It is likely that the rate of undetected RT in clinical practice, is at least as high as the one described based on this analysis, suggesting that this might continue to be an under-recognized phenomenon.

The nature of misattribution of RT to other phenomena is of clinical interest. The majority of cases of misattributed RT in our study were labeled as double triggering or simply ‘breath stacking’. Other cases were labeled as premature cycling or ineffective trigger. It is important for clinicians to be aware of these potential diagnostic errors, as the management can differ significantly, and misinterpretation could lead to an unnecessary increase in sedation in the patient who is asynchronous with the ventilator. For example, double triggering and RT with breath-stacking may appear similar, but in the former, the initial breath is patient triggered, and the respiratory effort is sufficient and prolonged enough to trigger a second ventilator delivered breath and leading to breath stacking. This is primarily induced by a patient’s high respiratory drive and failure of the ventilator to provide sufficient support compared to the demand, and deeper sedation is one of the options to control it. In contrast, patients’ effort seen in RT are induced by mechanical insufflation by the ventilator in patients who are deeply sedated and an option is to reduce sedation to let the patient takes control of the ventilator [5]. RT can also lead to breath-stacking if it occurs late enough in the respiratory cycle, and the patient effort is sufficient to trigger a second ventilator delivered breath. Observational studies have demonstrates that up to 35% of breath-stacking is due to RT [79]. It is important to differentiate breath-stacking secondary to double triggering versus RT, as they may require different approaches to management [5].

The clinical impact of RT is not fully understood. It has been linked to diaphragm dysfunction [7], increased tidal volumes in pressure-regulated modes of ventilation [8] and loss of lung protective ventilation due to breath stacking [10]. In an editorial of the ROSE trial, Slutsky et al. hypothesized that undetected RT in the deeply sedated patients of the ACURASYS trial may have contributed to worse outcomes in this group (due to consequent breath stacking and loss of lung protective ventilation) [80]. Similarly, the investigators of the Alveolar Recruitment trial suggest that undetected breath stacking may have occurred and contributed to worse outcomes in the treatment arm, as these patients were managed with higher PEEP and were likely to lose lung protective ventilation due to higher airway pressures in the event of breath stacking [81]. An increased awareness of RT may lead to a better management and a more targeted approach to management of asynchrony and breath stacking.

This review demonstrates that the majority of cases of published examples of RT occurred after the introduction of low tidal volume ventilation [14]. The observed temporal relation between the introduction of low tidal volume ventilation and the appearance of reverse triggering is not a definitive association but is of interest given the findings that reverse triggering may be associated with small volume ventilation strategy [5, 7, 11, 12]. While low tidal volume ventilation is clinically important, it has been argued that it primarily provides mortality benefit in patients with reduced respiratory system elastance [82].and it could be a modifiable risk factor for reverse triggering in the correct patient population.

### Strengths and limitations

Our study has strengths. It is the first systematic review of ventilator waveforms that examines the frequency of published undetected cases of RT, and where relevant, the alternative mechanism that was attributed to the observed asynchrony. It also used a robust methodology and confrontation with independent experts.

Our study also has limitations. Although we used a rigorous and reproducible literature search, accounted for duplicates, and had clear inclusion and exclusion criteria, we did not pre-publish our review protocol since such an approach with figures has never been described before. Furthermore, our search included only papers with specific mention of asynchronies and therefore would have missed published ventilator waveforms in other literature that may have missed RT events. Some of the papers included in our review were not published with the intention of identifying or naming specific ventilator asynchronies and therefore it could be argued that we would not expect authors to ‘detect’ RT in these cases. Furthermore, the number of eligible waveforms in each study was highly variable, introducing the potential for bias. The analysed waveforms were felt to be of sufficient quality to determine the type of breath and figure legends often contained information related to the triggering nature of the breaths, but we based our diagnosis on small, published images and very short recordings. The longer the duration of the waveform, the easier it is to identify entrainment, which was most of the time impossible. It is also increasingly recognized that the pattern of respiratory entrainment in RT is modifiable by numerous factors and may appear irregular [5]. Furthermore, we excluded spontaneous modes of ventilation in our study. While RT has been described in the literature in assisted modes of ventilation, for instance, secondary to auto-cycling [83]. it should be substantially more common in controlled or assist-control modes of ventilation. As such, this is where we directed our search and subsequent analysis. Finally, while it was not a primary objective of this study, the clinical details of the cases associated with the included waveforms was either incomplete or absent in many cases.

## Conclusion

RT is a common asynchrony, with a high rate of undetected RT in the published literature in the era of protective lung ventilation, reflecting the fact that it may also be frequently undetected in clinical practice as well. While diagnosis in the literature has improved in cases with definitive monitoring by Pes or EAdi, it continues to be undetected when these techniques are not used, identifying a potential area for education and quality improvement in the care of the mechanically ventilated patient.

### Electronic supplementary material

Below is the link to the electronic supplementary material.

Supplementary Material 1

Supplementary Material 2

Supplementary Material 3

Supplementary Material 4

## Data Availability

The datasets used and/or analysed during the current study are available from the corresponding author on reasonable request.

## Abbreviations

***EAdi***: *Electrical activity of the diaphragm*. A monitoring tool that enables measurement of electrical depolarisation of the diaphragm
***Pes***: *Esophageal pressure*. A monitoring tool that measures esophageal pressure changes during the respiratory cycle
***RT***: *Reverse triggering*. A type of patient-ventilator asynchrony defined as a patient inspiratory effort occurring after the onset of mechanical insufflation, appearing to be triggered by the ventilator’s insufflation
