# Supplementary material for: Reverse triggering ? a novel or previously missed phenomenon?
Source: Ann Intensive Care. 2024 May 22;14:78. doi: 10.1186/s13613-024-01303-4 (PMC11111438; doi:10.1186/s13613-024-01303-4)
Supplement: Supplementary file 1 — Supplementary Material 1 [file 13613_2024_1303_MOESM1_ESM.docx]

**Abstract word count**: 287

**Manuscript word count**: 3294

# Reverse Triggering – A Novel or Previously Missed Phenomenon?

Robert Jackson, MBChB^1,2^, Audery Kim, RN^1^, Nikolay Moroz, MD RRT^1,3^, L. Felipe Damiani, PhD^1,2,4^, Domenico Luca Grieco, MD^1,2,5,6^, Thomas Piraino, RRT^7^, Jan O. Friedrich MD DPhil^1,2^, Alain Mercat MD PhD^8^, Irene Telias, MD*^1,2^, Laurent Brochard, MD*^1,2^

*Co-senior authors

**Corresponding author**: Laurent J. Brochard, M.D., Li Ka Shing Knowledge Institute, 209 Victoria Street, Room 4-08, Toronto, ON, M5B 1T8 Canada. [laurent.brochard@unityhealth.to](mailto:laurent.brochard@unityhealth.to)

**Affiliations**

1. Keenan Centre for Biomedical Research, Li Ka Shing Knowledge Institute and St. Michael’s Hospital, Unity Health Toronto, Toronto, Ontario, Canada
2. Interdepartmental Division of Critical Care Medicine, University of Toronto, Toronto, Ontario, Canada
3. Department of Respiratory Therapy, McGill University Health Centre, Montreal, Quebec
4. Departamento Ciencias de la Salud, Carrera de Kinesiología, Facultad de Medicina, Pontificia Universidad Católica de Chile, Santiago, Chile
5. Department of Anesthesiology and Intensive Care Medicine, Catholic University of the Sacred Heart, Rome, Italy; Anesthesia
6. Emergency and Intensive Care Medicine, Fondazione Policlinico Universitario A. Gemelli IRCCS, Rome, Italy.
7. Department of Anesthesia, Division of Critical Care, McMaster University, Hamilton, Ontario, Canada
8. Medical ICU and Vent'Lab, University Hospital of Angers, University of Angers, 4 Rue Larrey, 49933, Angers Cedex 9, France

**Prior Abstract:** Reverse Triggering, A Missed Phenomenon in the Literature. Critical Care Canada Forum 2019 Abstracts. *Can J Anesth/J Can Anesth* **67** (Suppl 1), 1–162 (2020). https://doi-org.myaccess.library.utoronto.ca/10.1007/s12630-019-01552-z

**Declarations**

- Ethics approval and consent to participate: Not applicable.
- Consent for publication: Not applicable.
- Availability of data and material: The datasets used and/or analysed during the current study are available from the corresponding author on reasonable request.
- Competing interests: LB'S laboratory received research grants from Medtronic, Draeger, Stmit and Vitalaire and equipment from Sentec, Cerebra Health, Philips, Fisher Paykel. The remaining authors declare that they have no relevant competing interests.
- Funding: Not applicable.
- Authors' contributions: RJ had full access to all the data in the study and takes responsibility for the integrity of the data and the accuracy of the data analysis. RJ, AK, NM, IT and LB completed data analysis and interpretation. RJ, LFD, DLG, TP, JF, AM, IT and LB contributed substantially to the study design, data analysis and interpretation, and the writing of the manuscript.
- Acknowledgements: Not applicable.

## Key Words List

Lung Protective Ventilation, Mechanical Ventilation, Reverse Triggering, Ventilator Asynchrony.

## Abbreviations List

***EAdi****: Electrical activity of the diaphragm*. A monitoring tool that enables measurement of electrical depolarisation of the diaphragm.
***Pes****: Esophageal pressure*. A monitoring tool that measures esophageal pressure changes during the respiratory cycle.
***RT****: Reverse triggering*. A type of patient-ventilator asynchrony defined as a patient inspiratory effort occurring after the onset of mechanical insufflation, appearing to be triggered by the ventilator’s insufflation.

## Abstract

**Background**: Reverse triggering (RT) was described in 2013 as a form of patient-ventilator asynchrony, where patient’s respiratory effort follows mechanical insufflation. Diagnosis requires esophageal pressure (P_es_) or diaphragmatic electrical activity (EA_di_), but RT can also be diagnosed using standard ventilator waveforms.

**Hypothesis:** We wondered 1) how frequently RT would be present but undetected in the figures from literature, especially before 2013 ; 2) whether it would be more prevalent in the era of small tidal volumes after 2000.

**Methods:** We searched PubMed, EMBASE, and the Cochrane Central Register of Controlled Trials, from 1950 to 2017, with key words related to asynchrony to identify papers with figures including ventilator waveforms expected to display RT if present. Experts labelled waveforms. ‘Definite’ RT was identified when P_es_ or EA_di_ were in the tracing, and ‘possible’ RT when only flow and pressure waveforms were present, Expert assessment was compared to the author’s descriptions of waveforms.

**Results:** We found 65 appropriate papers published from 1977 to now, containing 181 ventilator waveforms. 21 cases of ‘possible’ RT and 25 cases of ‘definite’ RT were identified by the experts. 18.8% of waveforms prior to 2013 had evidence of RT. Most cases were published after 2000 (1 before vs 45 after, p=0.03). 54% of RT cases were attributed to different phenomena. A few cases of identified RT were already described prior to 2013 using different terminology (earliest in 1997). While RT cases attributed to different phenomena decreased after 2013, 60% of ‘possible’ RT remained missed.

**Conclusion:** RT has been present in the literature as early as 1997, but most cases were found after the introduction of low tidal volume ventilation in 2000. Following 2013, the number of undetected cases decreased, but RT are still commonly missed.

## Introduction

Patient ventilator asynchronies are common and have been associated with longer duration of mechanical ventilation^1,2^ and higher mortality^3^. The impact and prevalence of asynchrony is often under-estimated given challenges in identification ^4^.

Reverse triggering (RT) is a form of asynchrony described by Akoumanaki et al in 2013, occurring in up to 30-55% of sedated patients on controlled or assist-control ventilation^5^RT is defined as a patient inspiratory effort occurring after the onset of mechanical insufflation, appearing to be triggered by the ventilator’s insufflation^6^. While the mechanisms leading to RT are not fully understood, a frequent mechanism is that of respiratory entrainment; the establishment of a fixed, repetitive rhythm between a patients respiratory pattern generator and an external stimulus (here mechanical insufflation)^5^. . The physiological consequences of RT are not yet known. In an animal model, only when the respiratory effort was large, RT was associated with diaphragm dysfunction^7^. RT can increase tidal volumes in pressure-regulated modes of ventilation^8^.The reverse triggered patient effort may be significant enough to trigger a second breath before complete exhalation, also known as breath stacking^9^. This appears similar to double triggering, where a patients effort triggers the first ventilator breath and is sustained long enough to trigger a second breath after the ventilator cycles off. Both reverse triggering and double triggering can lead to injurious ventilation due to breath stacking, albeit via distinct underlying mechanisms^10^. Therefore, RT can be present without (most frequently) or with breath-stacking. The circumstances facilitating RT are not fully understood but have been associated with sedation ^10^ and low tidal volume ventilation^5,11,12^. In experimental models RT could be easily induced using reduced tidal volume ^7^.

The gold standard for diagnosis uses a direct measure of patient respiratory muscle activity, e.g., diaphragmatic electrical activity (EA_di_) or esophageal pressure (P_es_). RT is diagnosed when the patient’s respiratory effort is initiated after the start of mechanical insufflation**.** Frequently, this may occur in a regular, recurring pattern, suggesting that the patient’s respiratory cycle is ‘entrained’ to the set rate of the ventilator, but this may not always be the case and the pattern can appear irregular^5^. In the absence of such monitoring, RT can still be detected in the flow and pressure ventilator waveform characteristic of RT^13^ (Fig. 1); these changes are sometimes subtle and may be easily missed.

While RT was only recent described in critically ill patients^6^**,** it is likely that this phenomenon was already present and often missed. There is no large historical database of recordings that would allow measurement of the real burden of this phenomenon retrospectively, however there are publications including respiratory waveforms from which it may be possible to estimate the historical burden of this phenomenon. We wondered whether 1) RT in earlier publications may have gone undetected, with or without attribution to another phenomenon, 2) RT may still be undetected and/or attributed to another type of asynchrony in the absence of direct monitoring of the onset of patient effort. 3) the novelty of this phenomenon may also be related to the era of lung protective ventilation which was generalized mostly after 2000^14^. In this study we conducted a systematic review of the literature to identify the prevalence of undetected RT (either attributed to other phenomena or not) in previously published figures with ventilator waveforms. We analyzed cases prior to and after the initial description of RT in 2013, as well as before and after the introduction of low tidal volume ventilation in 2000, to examine any temporal relationship related to this change in ventilation strategy. Finally, we describe cases where RT was attributed to different phenomenon to better understand the nature of misattribution.

## Methods

**Eligibility Criteria**

We started from all previously published articles potentially describing ventilator waveforms in detail and where RT was expected to be possibly present and described if present, *i.e.*, those including patients on mechanical ventilation and discussing issues related to patient-ventilator asynchrony (see search strategy, supplementary figure 1). After abstract screening, when full texts were retrieved, we included all studies and supplementary material that contained a ventilator waveform (with at least flow and airway pressure waveforms present, with or without Pes or EAdi) from a patient on any mode of controlled or assist-control ventilation, with a minimum recording of two consecutive breaths. The terms controlled ventilation and assist-control ventilation are interchangeably used in the literature but we were looking for mandatory pre-determined breaths triggered or not by the patient. Manuscripts were excluded if studies involved only animal, neonate or pediatric patient population, negative pressure ventilation, high frequency oscillation or spontaneous modes of ventilation (i.e., partial assistance like pressure support ventilation).

**Database Search**

We searched Medline, EMBASE and the Cochrane Central Register of Controlled Trials for eligible articles between 1950-2017, using keywords related to asynchrony. Our search strategy is listed in the supplement (Supplementary Figure 1). Four authors (IT, LFD, DLG, TP) independently screened eligible abstracts using Covidence® systematic review tool.

**Data Collection Process**

Articles were then scanned for eligible figures. Figures were analyzed independently by three authors (RJ, AK, NM). Figures from eligible manuscripts were excluded if there was insufficient information on the figure (single breath recording, isolated waveform of flow or airway pressure), waveforms from animals, test lungs, simulated patients, neonates or pediatric patients, schematic representations, or from spontaneous modes of ventilation (i.e., pressure support, neurally adjusted ventilatory assist, proportional assist ventilation, synchronised intermittent mandatory ventilation). Following application of exclusion criteria, all included figures contained a waveform from a human patient ventilated in assist-control or controlled mode of ventilation. These will be subsequently referred to as “waveforms” for consistency.

Waveforms from assist-control mechanical ventilation modes were classified as those with spontaneous effort (when the breath is triggered by patient effort) or mandatory breaths (where there was no patient effort and the machine triggers the breath). All figure legends were reviewed for mention of reprint and duplicate waveforms were excluded.

**Waveform analysis**

Each waveform constituted our unit of analysis. Mandatory breath tracings were categorized into those with no identified RT, with *‘possible’ RT* (Paw and Flow waveforms demonstrating changes consistent with RT), or with *‘definite’ RT* (Pes or Eadi showing a patient inspiratory effort starting after mechanical ventilation) (Fig. 1).

*RT* was defined as evidence of onset of patient effort beginning after the initiation of a mandatory breath by the ventilator. The patient effort in RT should be temporally related to the onset of mechanical insufflation. Given that not all figures included a time-scale, it was not possible to use an absolute cut off. Instead, if the patient effort was visually within the first 50% of the whole respiratory cycle, it was considered to be RT, as opposed to ineffective effort.

*‘Definite RT’* was used where a method such as esophageal pressure or electrical activity of the diaphragm monitoring were used to directly demonstrate patient effort occurring after onset of mechanical insufflation.

*‘Possible RT’* was used where a patient effort beginning after the onset of mechanical insufflation was evident from changes in the flow and pressure waveforms from the mechanical ventilator. The criteria to diagnose ‘*possible RT’* were as follows:

1. Evidence of a mandatory breath delivered by the ventilator. Determined by:
   1. The absence of a negative deflection in the pressure-time curve immediately preceding the breaths.
   2. Indication in the text or figure legend as to whether breaths were mandatory or patient initiated.

AND at least one of:

1. Breath stacking following the mandatory breath.
2. Changes in the expiratory flow wave-form suggesting patient effort.
   1. A reduction in peak expiratory flow compared to other breaths.
   2. A positive deflection in expiratory flow disrupting the normal exponential decay of expiration.
3. Changes in the inspiratory phase
   1. during volume-control ventilation.
      1. a negative deflection in airway pressure.
   2. during pressure control ventilation.
   3. a negative deflection in airway pressure.

a positive deflection in inspiratory flow on the flow-time curve. Expert assessment was compared to the author’s descriptions of the waveforms either in the figure legends or the text. Waveforms with evidence of RT were labelled as *‘undetected’* if the figure legend or text did not describe RT or attributed the event to a different phenomenon. Disagreement was resolved by consensus , and all waveforms with RT were double-checked by the same authors (IT, LB).

**Additional data extraction**

Clinical data were obtained for cases of RT where available, either from figure legends or the manuscript where applicable.

**Outcomes**

Our primary outcome was the incidence of undetected cases of RT in the previously published literature, before and after the date of initial description in 2013. Secondary outcomes included a) the proportion of undetected cases in the subgroups of ‘possible’ and ‘definite’ RT, b) a summary of the terms used to describe undetected cases in the figure legends, and c) comparison of prevalence of ‘possible’ and ‘definite’ RT before and after 2000.

**Statistical analysis**

Analysis of the extracted waveforms was descriptive, including total number of identified cases of RT, and proportions of cases that were ‘definite’ versus ‘possible’. Categorical variables were analyzed using a chi squared (X2) test. Data analysis and creation of figures was done using Microsoft Excel (Version 2311).

## Results

**Study selection**

The search extracted 2700 articles and the selection process is described in Figure. 2. After applying our selection criteria, we identified 63 eligible articles for full review, and 2 articles were subsequently identified through an external search that comprised review of references from studies identified in the initial search. A total of 387 figures were assessed, with 206 figures excluded. Of these excluded figures, 17 out excluded as duplicates from other included articles. In total, 181 eligible waveforms were identified for analysis.

**Study/Waveform** **Characteristics**

In total, 65 eligible articles were included in this study. The publication dates ranged from 1977 to 2017 (Fig. 3). These included literature reviews, case reviews/series, prospective clinical studies, prospective observational studies, diagnostic testing studies and a prospective randomised clinical trial. *Table 1* describes the study characteristics and breakdown of included waveforms ^1,4,6,15–78^. Where available, clinical details for cases of RT can be found in *Table 2.*

**Waveform analyses and undetected RTs**

We retrieved 181 eligible waveforms published from 1977 to 2017 with 23 waveforms (12.7%) published prior to the year 2000 (Fig. 3). 46 waveforms (25.4%) showed evidence of RT, 45 being after 2000 (prevalence of RT 4% vs 28%, X^2^=4.6 , p=0.03). We classified 21 of these 46 as ‘possible’ RT and 25 as ‘definite’ RT (Fig. 4) Overall, there were 25 of the 46 waveforms with evidence of RT (54%) that were undetected. 17 out of 21 cases of ‘possible’ RT were undetected (80.1%) and 8 out of 25 cases of ‘definite’ RT were undetected (32%).

The figure legends for undetected cases of RT were extracted and collated in *Table 4*. ‘Double Triggering’ was the term most often mentioned in the description of the cases. The term breath-stacking was also often used, but in association with a mechanism other than reverse triggering.

**2013 timepoint and definitive versus possible RTs**

112 out of 181 waveforms were published pre-2013. In total, 21 out of 112 waveforms from 1997 to 2012 had evidence of RT (18.7%), with 11 ‘possible’ RT and 10 ‘definite’ RT. Most RT cases were not defined as RT (18 undetected RT cases pre-2013, 85.7% of total waveforms, Fig. 5). Three out of 10 ‘definite’ RT cases^24,31,37^ described the pattern and findings of RT in their respective figure legends without specifically naming it (as the phenomenon had not yet been named as RT) (Table 3). Of these cases, the description by Kallet et al in 2005^37^ is closest to the current definition of RT: “These waveforms reflect a common observation during lung-protective ventilation, whereby a ventilator-triggered breath stimulated the patient’s spontaneous breathing effort”.

Post 2013, there were 10 cases of ‘possible’ RT, of which 6 were undetected. 15 cases of ‘definite’ RT were found and only 1 was undetected (Fig. 5). The total number of undetected cases post-2013 was 7 (28% of total waveforms with identified RT in this period). Undetected cases dropped dramatically (Fig. 4).

## Discussion

In this systematic review of ventilator waveforms included in manuscripts related to ventilator asynchrony, RT was found to be present frequently in patients identified by the authors for having asynchrony on control or assist-control ventilation. A significant proportion of cases of RT were undetected or attributed to other phenomena. The recognition increased substantially after the 2013 publication, but especially when an additional physiological signal was available. The diagnosis remains challenging when using only the ventilator waveforms. The prevalence of RT has been much higher after 2000 in the low tidal volume era.

As expected, undetected cases were published prior to the first time the phenomenon was named RT in 2013 even in the presence of definitive monitoring such as Pes or EAdi. Interestingly the phenomenon was already described long before that publication without gaining much attention ^24,31,37^. In contrast, post-2013, almost all published waveforms that had definitive monitoring correctly identified the phenomenon and named it RT. In the absence of definitive monitoring, there continued to be a significant proportion of ‘possible’ RT that was undetected post-2013. Given the subtle changes in ventilator waveforms that differentiate RT, it is not surprising that it remains difficult to diagnose at the bedside in absence of definitive techniques. This suggests the potential benefit of monitoring esophageal pressure and/or EAdi in the diagnosis and management of asynchronies or the need for automated methods^13^. Often reverse triggering was mistaken for double triggering and “breath stacking”. There is no direct evidence that monitoring techniques can reduce asynchrony during controlled or assist-control modes of ventilation, but a direct monitor could theoretically improve differentiation between these distinct phenomena, which is the first step towards effective management.

While we are not able to estimate the true prevalence of RT based on this study, it is of clinical interest that the rate of undetected RT remains relatively high even amongst the relatively recently published waveforms related to ventilator asynchrony. It is likely that the rate of undetected RT in clinical practice, is at least as high as the one described based on this analysis, suggesting that this might continue to be an under-recognized phenomenon.

The nature of misattribution of RT to other phenomena is of clinical interest. The majority of cases of misattributed RT in our study were labeled as double triggering or simply ‘breath stacking’. Other cases were labeled as premature cycling or ineffective trigger. It is important for clinicians to be aware of these potential diagnostic errors, as the management can differ significantly, and misinterpretation could lead to an unnecessary increase in sedation in the patient who is asynchronous with the ventilator. For example, double triggering and RT with breath-stacking may appear similar, but in the former, the initial breath is patient triggered, and the respiratory effort is sufficient and prolonged enough to trigger a second ventilator delivered breath and leading to breath stacking. This is primarily induced by a patient’s high respiratory drive and failure of the ventilator to provide sufficient support compared to the demand, and deeper sedation is one of the options to control it. In contrast, patients’ effort seen in RT are induced by mechanical insufflation by the ventilatorI in patients who are deeply sedated and an option is to reduce sedation to let the patient takes control of the ventilator ^5^. RT can also lead to breath-stacking if it occurs late enough in the respiratory cycle, and the patient effort is sufficient to trigger a second ventilator delivered breath. Observational studies have demonstrates that up to 35% of breath-stacking is due to RT ^79^. It is important to differentiate breath-stacking secondary to double triggering versus RT, as they may require different approaches to management^5^.

The clinical impact of RT is not fully understood. It has been linked to diaphragm dysfunction^7^, increased tidal volumes in pressure-regulated modes of ventilation^8^ and loss of lung protective ventilation due to breath stacking^10^. In an editorial of the ROSE trial, Slutsky et al hypothesized that undetected RT in the deeply sedated patients of the ACURASYS trial may have contributed to worse outcomes in this group (due to consequent breath stacking and loss of lung protective ventilation)^80^. Similarly, the investigators of the Alveolar Recruitment trial suggest that undetected breath stacking may have occurred and contributed to worse outcomes in the treatment arm, as these patients were managed with higher PEEP and were likely to lose lung protective ventilation due to higher airway pressures in the event of breath stacking^81^. An increased awareness of RT may lead to a better management and a more targeted approach to management of asynchrony and breath stacking.

This review demonstrates that the majority of cases of published examples of RT occurred after the introduction of low tidal volume ventilation^14^. The observed temporal relation between the introduction of low tidal volume ventilation and the appearance of reverse triggering is not a definitive association but is of interest given the findings that reverse triggering may be associated with small volume ventilation strategy ^12^, ^5,7,11^. While low tidal volume ventilation is clinically important, it has been argued that it primarily provides mortality benefit in patients with reduced respiratory system elastance^82^ and it could be a modifiable risk factor for reverse triggering in the correct patient population.

**Strengths and limitations.**

Our study has strengths. It is the first systematic review of ventilator waveforms that examines the frequency of published undetected cases of RT, and where relevant, the alternative mechanism that was attributed to the observed asynchrony. It also used a robust methodology and confrontation with independent experts.

Our study also has limitations. Although we used a rigorous and reproducible literature search, accounted for duplicates, and had clear inclusion and exclusion criteria, we did not pre-publish our review protocol since such an approach with figures has never been described before. Furthermore, our search included only papers with specific mention of asynchronies and therefore would have missed published ventilator waveforms in other literature that may have missed RT events. Some of the papers included in our review were not published with the intention of identifying or naming specific ventilator asynchronies and therefore it could be argued that we would not expect authors to ‘detect’ RT in these cases. Furthermore, the number of eligible waveforms in each study was highly variable, introducing the potential for bias. The analysed waveforms were felt to be of sufficient quality to determine the type of breath and figure legends often contained information related to the triggering nature of the breaths, but we based our diagnosis on small, published images and very short recordings. The longer the duration of the waveform, the easier it is to identify entrainment, which was most of the time impossible. It is also increasingly recognized that the pattern of respiratory entrainment in RT is modifiable by numerous factors and may appear irregular^5^. Furthermore, we excluded spontaneous modes of ventilation in our study. While RT has been described in the literature in assisted modes of ventilation, for instance, secondary to auto-cycling^83^, it should be substantially more common in controlled or assist-control modes of ventilation. As such, this is where we directed our search and subsequent analysis. Finally, while it was not a primary objective of this study, the clinical details of the cases associated with the included waveforms was either incomplete or absent in many cases.

## Conclusion

RT is a common asynchrony, with a high rate of undetected RT in the published literature in the era of protective lung ventilation, reflecting the fact that it may also be frequently undetected in clinical practice as well. While diagnosis in the literature has improved in cases with definitive monitoring by Pes or EAdi, it continues to be undetected when these techniques are not used, identifying a potential area for education and quality improvement in the care of the mechanically ventilated patient.

## References

1. Thille AW, Rodriguez P, Cabello B, Lellouche F, Brochard L. Patient-ventilator asynchrony during assisted mechanical ventilation. *Intensive Care Med* 2006;32(10):1515–1522.

2. Vaporidi K, Babalis D, Chytas A, et al. Clusters of ineffective efforts during mechanical ventilation: impact on outcome. *Intensive Care Med* 2017;43(2):184–191.

3. Blanch L, Villagra A, Sales B, et al. Asynchronies during mechanical ventilation are associated with mortality. *Intensive Care Med* 2015;41(4):633–641.

4. Dres M, Rittayamai N, Brochard L. Monitoring patient-ventilator asynchrony. *Curr Opin Crit Care* 2016;22(3):246–253.

5. Rodrigues A, Telias I, Damiani LF, Brochard L. Reverse Triggering during Controlled Ventilation: From Physiology to Clinical Management. *Am J Respir Crit Care Med* 2023;207(5):533–543.

6. Akoumianaki E, Lyazidi A, Rey N, et al. Mechanical Ventilation-Induced Reverse-Triggered Breaths. *Chest* 2013;143(4):927–938.

7. Damiani LF, Engelberts D, Bastia L, et al. Impact of Reverse Triggering Dyssynchrony during Lung-Protective Ventilation on Diaphragm Function: An Experimental Model. *Am J Respir Crit Care Med* 2022;205(6):663–673.

8. Baedorf Kassis E, Su HK, Graham AR, Novack V, Loring SH, Talmor DS. Reverse Trigger Phenotypes in Acute Respiratory Distress Syndrome. *Am J Respir Crit Care Med* 2021;203(1):67–77.

9. Beitler JR, Sands SA, Loring SH, et al. Quantifying unintended exposure to high tidal volumes from breath stacking dyssynchrony in ARDS: the BREATHE criteria. *Intensive Care Med* 2016;42(9):1427–1436.

10. Yoshida T, Nakamura MAM, Morais CCA, Amato MBP, Kavanagh BP. Reverse Triggering Causes an Injurious Inflation Pattern during Mechanical Ventilation. *Am J Respir Crit Care Med* 2018;198(8):1096–1099.

11. Su HK, Loring SH, Talmor D, Baedorf Kassis E. Reverse triggering with breath stacking during mechanical ventilation results in large tidal volumes and transpulmonary pressure swings. *Intensive Care Med* 2019;45(8):1161–1162.

12. Rodriguez PO, Tiribelli N, Fredes S, et al. Prevalence of Reverse Triggering in Early ARDS. *Chest* 2021;159(1):186–195.

13. Pham T, Montanya J, Telias I, et al. Automated detection and quantification of reverse triggering effort under mechanical ventilation. *Crit Care* 2021;25(1):60.

14. Ventilation with Lower Tidal Volumes as Compared with Traditional Tidal Volumes for Acute Lung Injury and the Acute Respiratory Distress Syndrome. *New England Journal of Medicine* 2000;342(18):1301–1308.

15. Harboe S. Weaning from Mechanical Ventilation by means of Intermittent Assisted Ventilation I.A.V. Case Reports. *Acta Anaesthesiologica Scandinavica* 1977;21(3):252–256.

16. Popova LM, Moiseev AN. Mechanics of breathing during prolonged artificial ventilation. *Resuscitation* 1980;8(1):29–41.

17. Siegel JH, Stoklosa JC, Borg U, et al. Quantification of asymmetric lung pathophysiology as a guide to the use of simultaneous independent lung ventilation in posttraumatic and septic adult respiratory distress syndrome. *Ann Surg* 1985;202(4):425–439.

18. Tokioka H, Saito S, Kosaka F. Comparison of pressure support ventilation and assist control ventilation in patients with acute respiratory failure. *Intensive Care Med* 1989;15(6):364–367.

19. Imsand C, Feihl F, Perret C, Fitting JW. Regulation of inspiratory neuromuscular output during synchronized intermittent mechanical ventilation. *Anesthesiology* 1994;80(1):13–22.

20. Blackson T, Ciarlo J, Rizzo A. A case of patient-ventilator dyssynchrony caused by inadvertent PEEP. *Respiratory care* 1995;40(11):1144–1147.

21. Branson RD, Marlnrvrc NR. Dual-Control Modes of Mechanical Ventilation. *Respiratory Care* 1996;41:294–305.

22. Dick CR, Sassoon CS. Patient-ventilator interactions. *Clin Chest Med* 1996;17(3):423–438.

23. Chao DC, Scheinhorn DJ, Stearn-Hassenpflug M. Patient-Ventilator Trigger Asynchrony in Prolonged Mechanical Ventilation. *Chest* 1997;112(6):1592–1599.

24. Leung P, Jubran A, Tobin MJ. Comparison of assisted ventilator modes on triggering, patient effort, and dyspnea. *Am J Respir Crit Care Med* 1997;155(6):1940–1948.

25. Tobin MJ, Laghi F, Jubran A. Respiratory muscle dysfunction in mechanically-ventilated patients. *Molecular and Cellular Biochemistry* 1998;179:87–98.

26. Imanaka H, Nishimura M, Takeuchi M, Kimball WR, Yahagi N, Kumon K. Autotriggering caused by cardiogenic oscillation during flow-triggered mechanical ventilation: *Critical Care Medicine* 2000;28(2):402–407.

27. Babuška R, Alic L, Lourens MS, Verbraak AFM, Bogaard J. Estimation of respiratory parameters via fuzzy clustering. *Artificial Intelligence in Medicine* 2001;21(1–3):91–105.

28. Officer TM, Wheeler DR, Frost AE, Rodarte JR. Respiratory Control During Independent Lung Ventilation. *Chest* 2001;120(2):678–681.

29. Sassoon CSH, Foster GT. Patient-ventilator asynchrony. *Current Opinion in Critical Care* 2001;28–33.

30. Fontes M. Progress in mechanical ventilation: *Current Opinion in Anaesthesiology* 2002;15(1):45–51.

31. Kallet RH, Luce JM. Detection of Patient-Ventilator Asynchrony During Low Tidal Volume Ventilation, Using Ventilator Waveform Graphics. *Respiratory Care* 2002;47:183–5.

32. Younes M, Kun J, Webster K, Roberts D. Response of Ventilator-Dependent Patients to Delayed Opening of Exhalation Valve. *Am J Respir Crit Care Med* 2002;166(1):21–30.

33. Kondili E, Prinianakis G, Georgopoulos D. Patient–ventilator interaction. *British Journal of Anaesthesia* 2003;91(1):106–119.

34. Blanch L, Bernabe F, Lucangelo U. Measurement of Air Trapping, Intrinsic Positive End-Expiratory Pressure, and Dynamic Hyperinflation in Mechanically Ventilated Patients. *RESPIRATORY CARE* 2005;50(1).

35. Bonetto C, Calo M, Delgado M, Mancebo J. Modes of Pressure Delivery and Patient–Ventilator Interaction. *Respiratory Care Clinics of North America* 2005;11(2):247–263.

36. Dhand R. Ventilator Graphics and Respiratory Mechanics in the Patient With Obstructive Lung Disease. *RESPIRATORY CARE* 2005;50(2).

37. Kallet RH, Campbell AR, Dicker RA, Katz JA, Mackersie RC. Work of Breathing During Lung-Protective Ventilation in Patients With Acute Lung Injury and Acute Respiratory Distress Syndrome: A Comparison Between Volume and Pressure-Regulated Breathing Modes. *RESPIRATORY CARE* 2005;50(12).

38. Nilsestuen JO, Hargett KD. Using ventilator graphics to identify patient-ventilator asynchrony. *Respir Care* 2005;50(2):202–234; discussion 232-234.

39. Georgopoulos D, Prinianakis G, Kondili E. Bedside waveforms interpretation as a tool to identify patient-ventilator asynchronies. *Intensive Care Med* 2006;32(1):34–47.

40. Kallet RH, Campbell AR, Dicker RA, Katz JA, Mackersie RC. Effects of tidal volume on work of breathing during lung-protective ventilation in patients with acute lung injury and acute respiratory distress syndrome. *Critical Care Medicine* 2006;34(1):8–14.

41. Chen C-W, Lin W-C, Hsu C-H. Pseudo-double-triggering. *Intensive Care Med* 2007;33(4):742–743.

42. Thille AW, Brochard L. Promoting Patient-Ventilator Synchrony. *Clinical Pulmonary Medicine* 2007;14(6):350–359.

43. Thille A, Brochard L. Interactions patient–ventilateur. *Réanimation* 2007;16(1):13–19.

44. Thille AW, Brochard L. Double triggering during assisted mechanical ventilation: Is it a controlled, auto-triggered or patient-triggered cycle? Reply to C.-W. Chen. *Intensive Care Med* 2007;33(4):744–745.

45. Chen C-W, Lin W-C, Hsu C-H, Cheng K-S, Lo C-S. Detecting ineffective triggering in the expiratory phase in mechanically ventilated patients based on airway flow and pressure deflection: Feasibility of using a computer algorithm. *Critical Care Medicine* 2008;36(2):455–461.

46. Pohlman MC, McCallister KE, Schweickert WD, et al. Excessive tidal volume from breath stacking during lung-protective ventilation for acute lung injury. *Critical Care Medicine* 2008;36(11):3019–3023.

47. De Wit M, Miller KB, Green DA, Ostman HE, Gennings C, Epstein SK. Ineffective triggering predicts increased duration of mechanical ventilation. *Critical Care Medicine* 2009;37(10):2740–2745.

48. De Wit M, Pedram S, Best AM, Epstein SK. Observational study of patient-ventilator asynchrony and relationship to sedation level. *Journal of Critical Care* 2009;24(1):74–80.

49. Kondili E, Akoumianaki E, Alexopoulou C, Georgopoulos D. Identifying and relieving asynchrony during mechanical ventilation. *Expert Review of Respiratory Medicine* 2009;3(3):231–243.

50. Mellott KG, Grap MJ, Munro CL, Sessler CN, Wetzel PA. Patient-Ventilator Dyssynchrony: Clinical Significance and Implications for Practice. *Critical Care Nurse* 2009;29(6):41–55.

51. Unroe M, MacIntyre N. Evolving approaches to assessing and monitoring patient–ventilator interactions: *Current Opinion in Critical Care* 2010;16(3):261–268.

52. Colombo D, Cammarota G, Alemani M, et al. Efficacy of ventilator waveforms observation in detecting patient–ventilator asynchrony. *Critical Care Medicine* 2011;39(11):2452–2457.

53. De Wit M. Monitoring of Patient-Ventilator Interaction at the Bedside. *Respir Care* 2011;56(1):61–72.

54. Liao K-M, Ou C-Y, Chen C-W. Classifying Different Types of Double Triggering Based on Airway Pressure and Flow Deflection in Mechanically Ventilated Patients. *Respiratory Care* 2011;56(4):460–466.

55. Blanch L, Sales B, Montanya J, et al. Validation of the Better Care® system to detect ineffective efforts during expiration in mechanically ventilated patients: a pilot study. *Intensive Care Med* 2012;38(5):772–780.

56. Chacon E, Estruga A, Murias G, et al. Nurses’ Detection of Ineffective Inspiratory Efforts During Mechanical Ventilation. *American Journal of Critical Care* 2012;21(4):e89–e93.

57. Correger E. Interpretation of ventilator curves in patients with acute respiratory failure. :13.

58. Laghi F, Goyal A. Auto-PEEP in respiratory failure. *MINERVA ANESTESIOLOGICA* 2012;78(2).

59. Branson RD, Blakeman TC, Robinson BR. Asynchrony and Dyspnea. *Respir Care* 2013;58(6):973–989.

60. Chanques G, Kress JP, Pohlman A, et al. Impact of Ventilator Adjustment and Sedation–Analgesia Practices on Severe Asynchrony in Patients Ventilated in Assist-Control Mode. *Critical Care Medicine* 2013;41(9):2177–2187.

61. Murias G, Villagra A, Blanch L. Patient-ventilator dyssynchrony during assisted invasive mechanical ventilation. *MINERVA ANESTESIOLOGICA* 2013;79(4).

62. Richard JCM, Lyazidi A, Akoumianaki E, et al. Potentially harmful effects of inspiratory synchronization during pressure preset ventilation. *Intensive Care Med* 2013;39(11):2003–2010.

63. Akoumianaki E, Maggiore SM, Valenza F, et al. The Application of Esophageal Pressure Measurement in Patients with Respiratory Failure. *Am J Respir Crit Care Med* 2014;189(5):520–531.

64. Brochard L. Measurement of esophageal pressure at bedside: pros and cons. *Current Opinion in Critical Care* 2014;20(1):39–46.

65. Mellott KG, Grap MJ, Munro CL, et al. Patient ventilator asynchrony in critically ill adults: Frequency and types. *Heart & Lung* 2014;43(3):231–243.

66. Chiew YS, Pretty CG, Beatson A, et al. Automated logging of inspiratory and expiratory non-synchronized breathing (ALIEN) for mechanical ventilation [Internet]. In: 2015 37th Annual International Conference of the IEEE Engineering in Medicine and Biology Society (EMBC). Milan: IEEE; 2015 [cited 2023 Oct 8]. p. 5315–5318.Available from: https://ieeexplore.ieee.org/document/7319591/

67. Mietto C, Malbrain MLNG, Chiumello D. Transpulmonary pressure monitoring during mechanical ventilation: a bench-to-bedside review. *Anaesthesiol Intensive Ther* 2015;47(J):27–37.

68. Yonis H, Gobert F, Tapponnier R, Guérin C. Reverse triggering in a patient with ARDS. *Intensive Care Med* 2015;41(9):1711–1712.

69. Delisle S, Charbonney E, Albert M, et al. Patient–Ventilator Asynchrony due to Reverse Triggering Occurring in Brain-Dead Patients: Clinical Implications and Physiological Meaning. *Am J Respir Crit Care Med* 2016;194(9):1166–1168.

70. Figueroa-Casas JB, Montoya R. Effect of Tidal Volume Size and Its Delivery Mode on Patient–Ventilator Dyssynchrony. *Annals ATS* 2016;13(12):2207–2214.

71. Mauri T, Yoshida T, Bellani G, et al. Esophageal and transpulmonary pressure in the clinical setting: meaning, usefulness and perspectives. *Intensive Care Med* 2016;42(9):1360–1373.

72. Murias G, Haro C de, Blanch L. Does this ventilated patient have asynchronies? Recognizing reverse triggering and entrainment at the bedside. *Intensive Care Med* 2016;42(6):1058–1061.

73. Restrepo RD, Serrato DM, Adasme R. Assessing Respiratory System Mechanical Function. *Clinics in Chest Medicine* 2016;37(4):615–632.

74. Guervilly C, Bisbal M, Forel JM, et al. Effects of neuromuscular blockers on transpulmonary pressures in moderate to severe acute respiratory distress syndrome. *Intensive Care Med* 2017;43(3):408–418.

75. Ghedira S, Houissa M, Belkadi K, et al. Reverse triggering on mild and severe ARDS patients [Internet]. In: Annals of Intensive Care. 2017 [cited 2023 Oct 9]. p. 7.Available from: https://doi.org/10.1186/s13613-016-0224-7

76. Kamilia C, Regaieg K, Baccouch N, et al. Proceedings of Réanimation 2017, the French Intensive Care Society International Congress. *Annals of Intensive Care* 2017;7(1):7.

77. Sangha H, Whitacre T. Detection of Ventilator Autotriggering by an Esophageal Catheter Used to Monitor the Neural Input and Diaphragm Excitation. *J Intensive Care Med* 2017;32(2):170–173.

78. Tripathi M, Tripathi N, Pandey M. Asynchrony Between Ventilator Flow and Pressure Waveforms and the Capnograph on Dräger Anesthesia Workstations: A Case Report. *A & A Case Reports* 2017;8(5):122–125.

79. Haro C de, López-Aguilar J, Magrans R, et al. Double Cycling During Mechanical Ventilation: Frequency, Mechanisms, and Physiologic Implications. *Crit Care Med* 2018;46(9):1385–1392.

80. Slutsky AS, Villar J. Early Paralytic Agents for ARDS? Yes, No, and Sometimes. *N Engl J Med* 2019;380(21):2061–2063.

81. Writing Group for the Alveolar Recruitment for Acute Respiratory Distress Syndrome Trial (ART) Investigators, Cavalcanti AB, Suzumura ÉA, et al. Effect of Lung Recruitment and Titrated Positive End-Expiratory Pressure (PEEP) vs Low PEEP on Mortality in Patients With Acute Respiratory Distress Syndrome: A Randomized Clinical Trial. *JAMA* 2017;318(14):1335–1345.

82. Goligher EC, Costa ELV, Yarnell CJ, et al. Effect of Lowering tidal volume on Mortality in Acute Respiratory Distress Syndrome Varies with Respiratory System Elastance. *Am J Respir Crit Care Med* 2021;203(11):1378–1385.

83. Longhini F, Simonte R, Vaschetto R, Navalesi P, Cammarota G. Reverse Triggered Breath during Pressure Support Ventilation and Neurally Adjusted Ventilatory Assist at Increasing Propofol Infusion. *J Clin Med* 2023;12(14):4857.

## Figure Legends

**Figure 1.** Example tracings of waveforms demonstrating diagnostic features of reverse triggering (RT). RT can be diagnosed using only the ventilator waveform in either volume or pressure-controlled modes. The absence of negative deflection in the airway pressure waveform (*) indicates that these are mandatory breaths. **Figure 1A** demonstrates breath stacking after a mandatory breath in pressure control mode. The stacked breath following a passive mechanical insufflation due to a mandatory breath must be initiated by a patient effort, therefore this finding is consistent with RT. Additionally, the increase in flow towards the end of the breath (arrowhead) indicates patient effort). **Figure 1B** shows a mandatory volume-controlled breath with reverse triggering, followed by a mandatory breath without RT. There is a sudden decrease in expiratory flow (black arrow) a decrease in peak expiratory flow (dashed line, flow waveform) and plateau pressure (dashed line, pressure waveform), all corresponding to a patient effort. **Figure 1C** demonstrates a mandatory breath with reverse triggering in pressure control mode. The dotted line represents the anticipated morphology of a normal mandatory breath. There is an increase in end-inspiratory flow (arrowhead), a decrease in expiratory flow (arrow – flow waveform) and a subtle drop in peak pressure (arrow – pressure waveform) all representing patient effort.

The addition of esophageal pressure monitoring (*Pes*) provides a precise indicator of the delay between onset of mechanical insufflation and the beginning of patient effort (*vertical grey column*This is used to diagnose ‘**Definite RT’.**

**Figure 2.** Search Strategy. PAV = Proportional Assist Ventilation. NAVA = Neurally Adjusted Ventilatory Assist. PS=Pressure Support. SIMV = Synchronized Intermittent Mandatory Ventilation.

**Figure 3. Reverse triggering cases by year published** * = Cases published prior to 2013 that correctly describe reverse triggering without specifically naming it.

**Figure 4.** Breakdown of the categorization of included waveforms.

**Figure 5.** Reverse triggering (RT) cases detected by authors, by time period (pre and post initial description in 2013). **(A)** Cases of ‘Possible’ RT (i.e. without the use of Pes or EAdi) categorized as detected (by authors) or un-detected. Pre-2013, all cases of possible reverse triggering were misattributed. Post-2013 a significant proportion continued to be misattributed. **(B)** Cases of ‘Definite’ RT (i.e. using (Pes or Eadi), demonstrating that pre-2013 a select few cases correctly described the phenomenon before it’s definite description in 2013. Following 2013, most cases are detected when using definitive monitoring. *Pes: Esophageal pressure, EAdi: Electrical activity of the diaphragm*

**Table 1 -** A summary of details of papers from literature search included in the final analysis.

| Author | Year | Journal | Type of Article | Total waveforms included / Total Figures in Paper |
| --- | --- | --- | --- | --- |
| Harboe^15^ | 1977 | Acta Anaesthesiologica Scandinavica | Review | 3/3 |
| Popova^16^ | 1980 | Resuscitation | Case series | 4/4 |
| Siegel^17^ | 1985 | Annals of Surgery | Prospective clinical study | 2/15 |
| Tokioka^18^ | 1989 | Intensive Care Medicine | Prospective clinical study | 2/2 |
| Blackson^20^ | 1995 | Respiratory Care | Case study | 2/4 |
| Branson^21^ | 1996 | Respiratory Care | Review | 2/10 |
| Dick^22^ | 1996 | Clinics in Chest Medicine | Review | 2/4 |
| Chao^23^ | 1997 | Chest | Prospective Cohort | 3/3 |
| Leung^24^ | 1997 | American Journal of Respiratory and Critical Care Medicine | Prospective clinical study | 2/7 |
| Tobin^25^ | 1998 | Molecular and Cellular Biochemistry | Review | 1/12 |
| Imanaka^26^ | 2000 | Critical Care Medicine | Prospective Clinical Study | 1/2 |
| Babuska^27^ | 2001 | Artificial Intelligence in Medicine | Prospective Cohort, Physiologic Study | 1/6 |
| Officer^28^ | 2001 | Chest | Case study | 1/1 |
| Sassoon^29^ | 2001 | Current Opinion in Critical Care | Review | 2/8 |
| Fontes^30^ | 2002 | Current Opinion in Anaesthesiology | Review | 1/4 |
| Kallet^31^ | 2002 | Respiratory Care | Case study | 1/1 |
| Younes^32^ | 2002 | American Journal of Respiratory Critical Care Medicine | Prospective clinical study | 3/10 |
| Kondili^33^ | 2003 | British Journal of Anaesthesia | Review | 5/17 |
| Blanch^34^ | 2005 | Respiratory Care | Review | 4/17 |
| Bonetto^35^ | 2005 | Respiratory Care Clinics of North America | Review | 1/3 |
| Dhand^36^ | 2005 | Respiratory Care | Review | 1/16 |
| Kallet^37^ | 2005 | Respiratory Care | Prospective Clinical Study | 4/7 |
| Nilsestuen^38^ | 2005 | Respiratory Care | Review | 11/54 |
| Georgopoulos^39^ | 2006 | Intensive Care Medicine | Review | 6/61 |
|  |  |  |  |  |
| Kallet^40^ | 2006 | Critical Care Medicine | Prospective Clinical Study | 1/2 |
| Thille^1^ | 2006 | Intensive Care Medicine | Prospective cohort | 1/4 |
| Chen^41^ | 2007 | Intensive Care Medicine | Case Study / Correspondence | 1/1 |
| Thille^42^ | 2007 | Clinical Pulmonary Medicine | Review | 6/7 |
| Thille^43^ | 2007 | Réanimation | Review | 3/3 |
| Thille^44^ | 2007 | Intensive Care Medicine | Case / correspondence | 1/1 |
| Chen^45^ | 2008 | Critical Care Medicine | Prospective Clinical Study | 2/3 |
| Pohlman^46^ | 2008 | Critical Care Medicine | Prospective cohort | 2/4 |
| De Wit^47^ | 2009 | Critical Care Medicine | Prospective Cohort | 2/4 |
| De Wit^48^ | 2009 | Journal of Critical Care | Prospective cohort | 4/5 |
| Kondili^49^ | 2009 | Expert Review of Respiratory Medicine | Review | 1/19 |
| Mellot^50^ | 2009 | Critical Care Nurse | Review | 3/4 |
| Unroe^51^ | 2010 | Current opinion in Critical Care | Review | 1/4 |
| Colombo^52^ | 2011 | Critical Care Medicine | Prospective Observational | 3/4 |
| De Wit^53^ | 2011 | Respiratory Care | Review | 3/10 |
| Liao^54^ | 2011 | Respiratory Care | Prospective cohort | 3/4 |
| Blanch^55^ | 2012 | Intensive Care Medicine | Diagnostic Test Study | 3/8 |
| Chacón^56^ | 2012 | American Journal of Critical Care | Diagnostic Test Study | 1/1 |
| Correger^57^ | 2012 | Medicina Intensiva | Review | 4/24 |
| Laghi^58^ | 2012 | Minerva Anesthesiologica | Review | 2/14 |
| Akoumianaki^6^ | 2013 | Chest | Case Series | 6/9 |
| Branson^59^ | 2013 | Respiratory Care | Review | 4/6 |
| Chanques^60^ | 2013 | Critical Care Medicine | Prospective Cohort, Physiological Study | 3/7 |
| Murias^61^ | 2013 | Minerva Anaesthesiologica |  | 2/7 |
| Richard^62^ | 2013 | Intensive Care Medicine | Bench and prospective clinical study | 1/4 |
| Akoumianaki^63^ | 2014 | American Journal of Respiratory and Critical Care Medicine | Review | 3/6 |
| Brochard^64^ | 2014 | Current Opinion in Critical Care | Review | 1/2 |
| Mellot^65^ | 2014 | Heart & Lung | Prospective Observational | 12/24 |
| Chiew^66^ | 2015 | IEEE | Prospective Clinical Study | 1/3 |
| Mietto^67^ | 2015 | Anaesthesiology Intensive Therapy | Review | 3/5 |
| Yonis^68^ | 2015 | Intensive Care Medicine | Case report | 1/1 |
| Delisle^69^ | 2016 | American Journal of Respiratory and Critical Care Medicine | Case series / correspondence | 2/2 |
| Dres^4^ | 2016 | Current Opinion in Critical Care | Review | 2/3 |
| Figueroa-Casas^70^ | 2016 | Annals of the American Thoracic Society | Prospective clinical study | 6/9 |
| Mauri^71^ | 2016 | Intensive Care Medicine | Review | 6/10 |
| Murias^72^ | 2016 | Intensive Care Medicine | Review | 4/4 |
| Restrepo^73^ | 2016 | Clinics in Chest Medicine | Review | 2/22 |
| Guervilly^74^ | 2017 | Intensive Care Medicine | Prospective randomised controlled study | 2/5 |
| Mechati^76^ | 2017 | Annals of Intensive Care | Prospective observational | 2/2 |
| Sangha^77^ | 2017 | Journal of Intensive Care Medicine | Case Series | 1/7 |
| Tripathi^78^ | 2017 | A & A: Case Reports | Case Report | 5/5 |

**Table 2 –** A summary of clinical details related to waveforms that demonstrate reverse triggering.

| Author | Type of Study | Patients with RT (N) | Age, Years (Range) | Pathologies | Ventilator Mode (N) | PEEP (mean) | RR (mean) | Tidal Volume (mean) | Sedation Score | Pplat (or Pinsp) | PF ratio |
| --- | --- | --- | --- | --- | --- | --- | --- | --- | --- | --- | --- |
| Kallet, 2002 | Case Discussion | 1 | 55 | ARDS - Pneumonia, Septic Shock | VAC | 16 | 32 | 5 mL/kg |  | 30 | 77 |
| Kallet, 2005 | Prospective non-randomized trial | 2 | 39-72 | ARDS (Pneumonia, Sepsis), ALI- Polytrauma | VC (1), PCV (1) | 9.5 |  | 6 mL/kg |  |  | 150 (90-210) |
| Kallet, 2006 | Prospective non-randomized trial | 1 | 41 | ARDS - Sepsis | VC | 5 |  | 6 mL/kg | Ramsey 4 |  |  |
| Chen, 2007 | Case Report | 1 |  | Heart failure and pneumonia | PCV |  | 25 |  |  |  |  |
| Liao, 2011 | Prospective cohort | 12 | 43-89 | Sepsis (3), Pneumonia (4), COPD (3), VAP (1), CHF (1) | VC (10), PC (2) |  |  | 8.2 mL/kg | Ramsey 2 (1), Ramsey 3 (2). Ramsey 4 (1). Ramsey 5 (3), Coma (3) |  | 303 |
| Akoumianaki, 2013 | Retrospective Cohort | 8 | 25-73 | ARDS (Pneumonia, Sepsis, SIRS, Polytrauma) | PAC (3), VAC (5) | 10.7 | 22 | 6.8 mL/kg | RAS -4 to -5 | 26.1 | 161.9 |
| Chanques, 2013 | Physiological study | 2 | 66 | COPD | VC |  |  | 400 mL |  |  |  |
| Yonis, 2015 | Case Report | 1 | 50 | ARDS - Sepsis | VC | 13 | 24 | 340 mL | Ramsey 6 | 23 | 117 |
| Delisle, 2016 | Case study | 2 | 40-78 | Out of hospital cardiac arrest and Brain Death | VAC (2) | 5 | 15 | 480 mL |  |  |  |
| Murias, 2016 (ICM) | Review article | 4 |  | acute respiratory failure | VC (2), PC (2) | 6.75 | 17.5 | 397.75 mL | RAS 1 (3). RAS 4 (1) | 19.5 |  |
| Guervilly , 2017 | Prospective RCT | 1 |  | ARDS | VC |  |  | 6 mL/kg |  |  |  |
| Mechati, 2017 | Abstract - blinded RCT | 1 |  | ARDS |  |  |  |  |  |  |  |

There were 4 manuscripts with incomplete clinical details, including 5 waveforms demonstrating RT, all of which were seen during VCV. There were 17 manuscripts in which 20 figures demonstrated RT, but no relevant clinical data was described. Where blank, data was not reported.

*RT = Reverse Triggering, PEEP = Positive end expiratory pressure, RR=Set Respiratory Rate, Pplat = Plateua pressure, Pinsp = Peak Inspiratory Pressure, PF ratio = ratio of partial pressure of oxygen in arterial blood (PaO2) to the fraction of inspiratory oxygen concentration.*

**Table 3** – Figure legends from pre-2013 that identified RT based on an accurate description of the phenomenon.

| Article | Pre-2013 – Identified Reverse Triggering |
| --- | --- |
| **Leung et al, 1997** | ‘The final breath was machine-initiated, and patient effort commenced after the onset of flow delivery’ |
| **Kallet et al, 2002** | ‘Her inspiratory efforts appeared to commence at peak inspiration, and intermittently she was able to trigger extra mechanical breaths’ |
| **Kallet et al, 2005** | ‘These waveforms reflect a common observation during lung-protective ventilation, whereby a ventilator-triggered breath stimulated the patient’s spontaneous breathing effort.’ |

**Table 4** – Terms used in waveform descriptions in undetected ‘possible’ and ‘definite’ cases of reverse triggering.

| **‘POSSIBLE’ RT** | **Number of Mentions** |
| --- | --- |
| **Double Triggering** | 6 |
| **Breath Stacking** | 3 |
| **Inadequate Ventilator Flow** | 2 |
| **Ineffective Efforts** | 1 |
| **Premature Cycling** | 2 |
| **Trigger Dyssynchrony** | 1 |
| **Double Cycling** | 1 |
| **Other/No Mention** | 2 |
| **‘DEFINITE’ RT** | **Number of Mentions** |
| **Double Trigger** | 4 |
| **Ineffective Efforts** | 2 |
| **Pseudo-double triggering** | 1 |
| **Auto-triggering** | 1 |
| **Other/No Mention** | 1 |


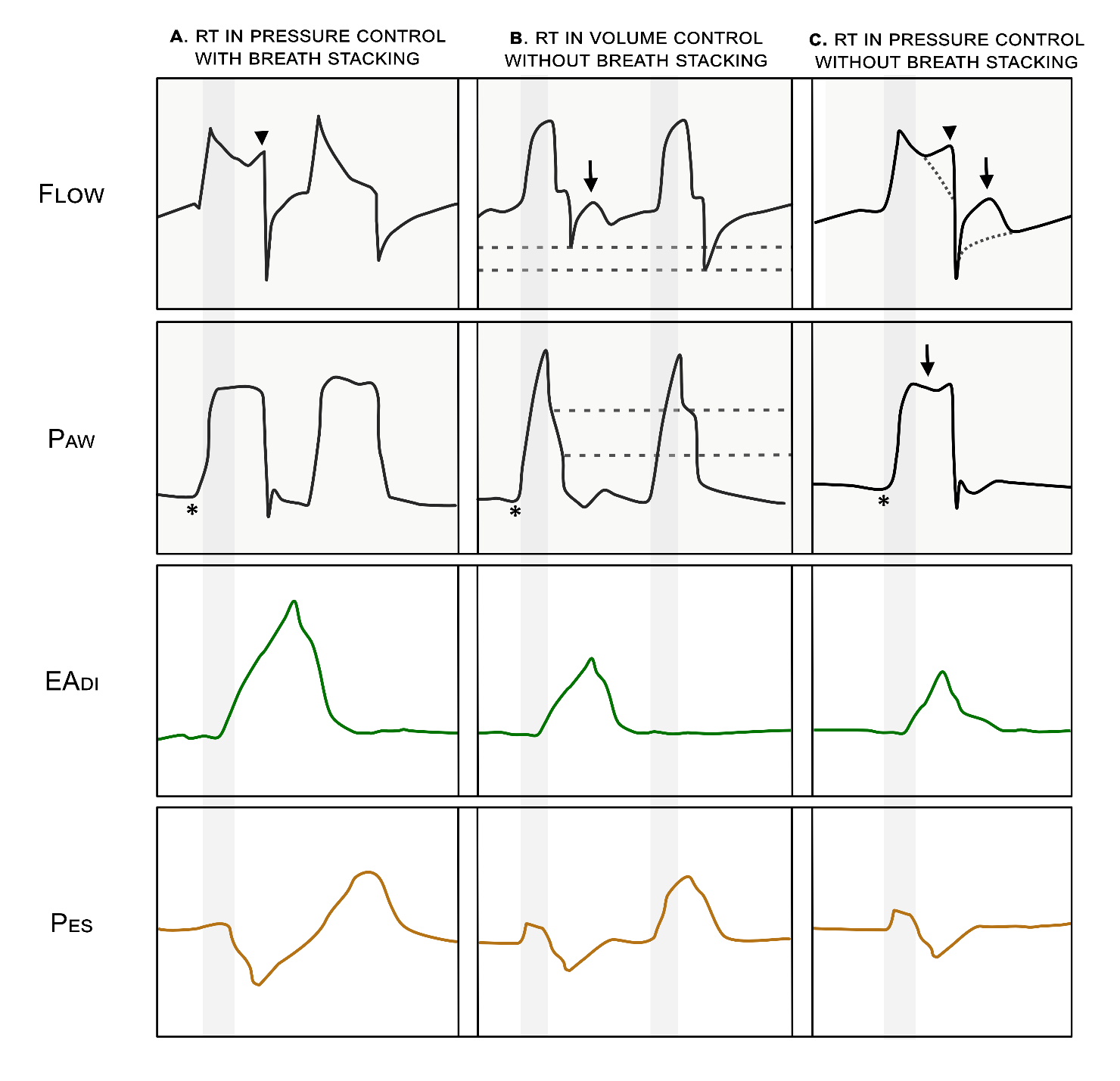


**Fig. 1**

Electronic Database Search: PubMed, EMBASE, and the Cochrane Central Register of Controlled Trials. 4158 Citations Identified.

2700 Abstracts screened.

963 Articles eligible for full-text screening.

63 Articles full-text screening.

1458 duplicates removed.

1737 titles and abstracts excluded for no relevance.

900 excluded for 1+ of: no respiratory waveforms, paper not accessible, or if waveforms in paper were only from neonates, infants, test lungs, animals, or purely spontaneous modes of ventilation.

2 Articles obtained through external search of references from identified articles.

65 Articles in total.
387 figures obtained and analyzed.

181 waveforms included and analysed.

206 figures excluded for non-invasive ventilation, pure spontaneous ventilation modes (PAV, NAVA, PS, *SIMV*), schematic representations or incomplete waveforms, waveforms from animals or test lungs. 17 of these excluded figures were removed as they were duplicates from other included studies.

**Fig. 2**


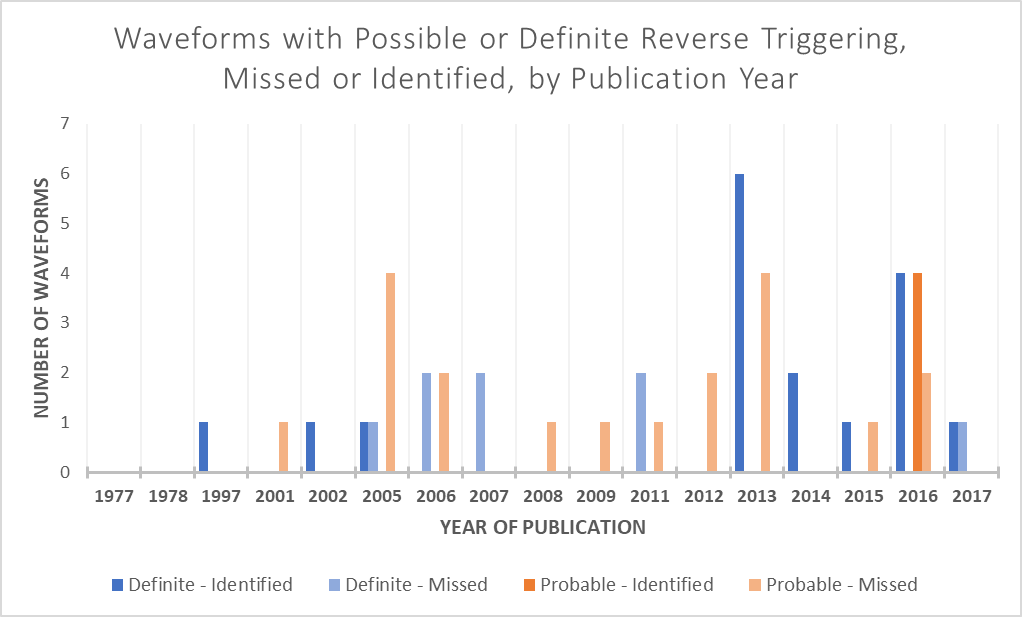


**Fig. 3**

*****

*****

*****


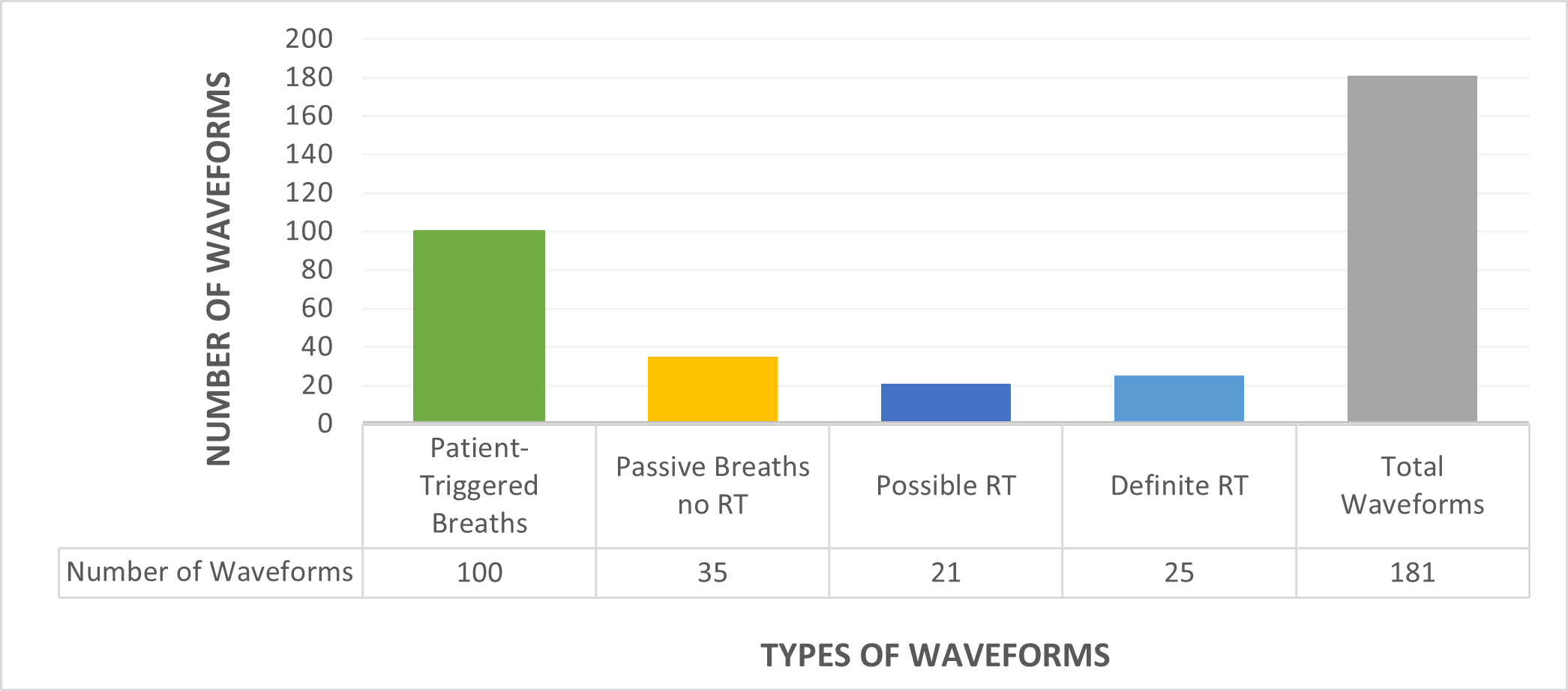


**Fig. 4**


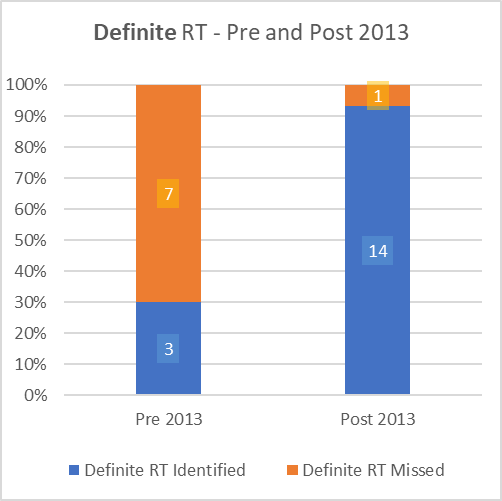

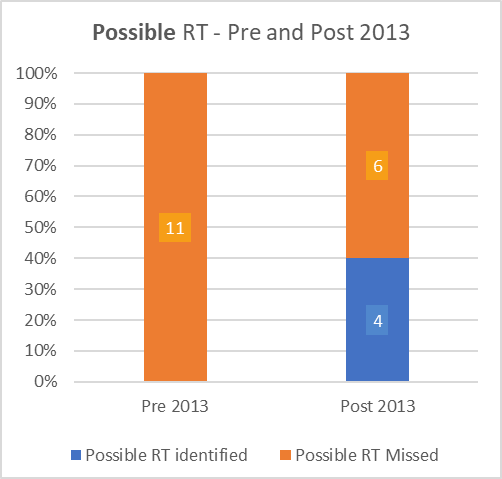


**Fig. 5**

**A**

**B**
