# Supplementary material for: Reverse triggering ? a novel or previously missed phenomenon?
Source: Ann Intensive Care. 2024 May 22;14:78. doi: 10.1186/s13613-024-01303-4 (PMC11111438; doi:10.1186/s13613-024-01303-4)
Supplement: Supplementary file 2 — Supplementary Material 2 [file 13613_2024_1303_MOESM2_ESM.docx]

# Supplementary Methods

# Supplementary Figure 1: Search Strategy and Results.

Numbers of papers in parenthesis; * = truncation term, adj = adjacent terms

**(A) WORDS RELATED TO ASYNCHRONY:** synchron* OR asynchron* OR dyssynchron* OR dys-synchrony OR double adj trigger* OR double adj cycl* OR breath adj stack* OR air adj stack* OR short adj cycl* OR premature adj cycl* OR premature adj term* OR long adj cycl* OR prolong* adj cycl* OR delayed adj cycl* OR delayed adj term* OR ineff* adj effort* OR wasted adj effort* OR reverse adj trigger* OR entrainment OR patient-ventilat* adj interaction OR patient adj ventilat* adj interaction OR patient adj ventilator adj synchrony (**254486**)

**AND**

**(B) WORDS RELATED TO MECHANICAL VENTILATION:** (artificial adj respiration OR mechanical* adj ventilat* OR ventilat*) **(456610)**

**A + B = C = 5828**

**C + RESTRICTION TO HUMAN DATA = 4158**

**Remove duplicates = 2700**

**LANGUAGE**

Any
