# Supplementary material for: Reverse triggering ? a novel or previously missed phenomenon?
Source: Ann Intensive Care. 2024 May 22;14:78. doi: 10.1186/s13613-024-01303-4 (PMC11111438; doi:10.1186/s13613-024-01303-4)
Supplement: Supplementary file 4 — Supplementary Material 4 [file 13613_2024_1303_MOESM4_ESM.docx]

# Response to Reviewers: Reverse Triggering – A Novel or Previously Missed Phenomenon?

**Manuscript**: AOIC-D-24-00138

Dear Editors**,**

Thank you for allowing us the opportunity to submit a revised manuscript to Annals of Intensive Care. We appreciate the time that you and the reviewers have spent to provide this valuable feedback. We responded to all comments and have incorporated the majority of the suggestions that the reviewers made. These changes have been highlighted in the revised draft of the manuscript. Our point-by-point responses are below in blue text. We simplified and clarified the presentation and carefully rephrased some of our conclusions.

## Reviewer’s Comments to the Authors

### Reviewer #1:

Thank you for giving me the opportunity to review this manuscript.
In this research article on asynchrony and specifically on reverse triggering, the authors present a systematic review with figures analysis that has not been described before. With 66 (or 65?) articles reported and 181 respiratory waveforms analyzed, the authors aimed to determine the incidence of detected and undetected cases of reverse triggering before and after 2013 (date of initial description of the reverse triggering), before and after 2000 (date of transition from high to low tidal volume era, potential contributor to reverse triggering).

**GENERAL COMMENT:** This review is very well written and accurately describes reverse triggering and its implications. It's very interesting to see how descriptions have evolved over the years. However, we must be cautious in our interpretation, as it is difficult to determine the incidence and the impact of a phenomenon that was not accurately described before 2013 and therefore not investigated. Also, it is therefore speculative to establish a correlation between reverse triggering and lower tidal volume on the basis of this data.

We thank the reviewer for the valuable feedback. We agree with the comment that we must be cautious in our interpretation and modified our wording accordingly. One aim of our study was to determine if reverse triggering was present prior to 2013 and to estimate its incidence in the literature. It is very difficult to determine the real incidence of reverse triggering because until very recently no algorithm existed to detect it automatically, and very few studies yet have looked at it in a rigorous way recording waveforms over a sufficient period of time and labeling breath by breath all the recordings (a very time-consuming process that we have done a few times in our laboratory). We imagined this creative solution to test our broad hypothesis. These data are useful to explain whether reverse triggering is really a new phenomenon, when it first began to appear in the literature, and to make inferences based on the temporal association with changes in ventilator management.

The reviewer is correct that the association of low tidal volume ventilation and reverse triggering is speculative based on these data. Low tidal volumes have been suggested elsewhere to be a risk factor [1] and we thought this could be reflected in the "prevalence" over time. The identified articles in our search were most often narrative review articles (e.g. Georgopoulos et al)[2], in which no clinical data were provided regarding tidal volumes. We did the search in a systematic fashion to ensure we were not missing the data that would allow us to further interrogate this association. Unfortunately, as can be seen in table 2 in our manuscript, data regarding tidal volumes were most often absent or at best incomplete (for instance tidal volumes not normalised to ideal body weight).

In response to the reviewer’s comments, we have edited our conclusions to reflect the limitations of the methods of the paper as follows:

*“**The observed temporal relation between the introduction of low tidal volume ventilation and the appearance of reverse triggering is not a definitive association but is of interest given the findings that reverse triggering may be associated with small volume ventilation strategy.” (Page 14, Line 294)*

**MAJOR COMMENTS:**
1. Table 1: I don't think we should consider the article of Georgopoulos et al. (ICM 2006) and its supplemental as two different articles. I think it should be considered as a single article, so that in total the review would cover 65 and not 66 articles.

Thank you for pointing this out. We agree with the reviewer’s suggestion and have corrected this in our manuscript. All references to “66 articles” have been changed to “65 articles” *(see line 67, line 211 and figure 1),* and the respective rows for the Georgopoulos article and supplement in *table 1* have been merged.
**MINOR COMMENTS:**
1. Line 80: You described reverse triggering as a form of asynchrony occurring in patients under assist-control ventilation. Don't you think it could happen under a totally mandatory ventilation mode, after initiation of a mandatory breath (without breath stacking obviously)?

We agree with the reviewer’s comments that reverse triggering does occur primarily in mandatory modes of ventilation. In our manuscript we use the term assist-control to refer to the modes of ventilation in which reverse triggering occurs in our analysis, however a more accurate description would be “controlled or assist-control” modes. We have therefore updated the terminology throughout the manuscript accordingly (see line 81, line 126, line 259, line 321) and mentioned that assist-control and controlled ventilation are often used interchangeably:

*“The terms controlled ventilation and assist-control ventilation are interchangeably used in the literature, but we were looking for mandatory pre-determined breaths triggered or not by the patient” page 7, line 126*

2. And don't you think it could happen under pressure support ventilation (excluded in your review), after initiation of an auto-cycled breath?

The reviewer raises an interesting point. Reverse triggering by definition occurs after the initiation of a mandatory breath and is therefore more likely to occur during controlled or assist control ventilation and much less likely in pressure support when the patient should be triggering the ventilator. However, in the event that the patient for some reason did not trigger the ventilator and received an auto-cycled breath, or even an auto-triggered breath, this could in theory lead to reverse triggering. However, given the nature of transitioning patients to pressure support when they are making spontaneous efforts, we believe it would be much less common to see reverse triggering in this mode. Indeed a recent study by Longhini et al analysed the number of reverse triggered breaths seen in patients on pressure support ventilation and varying levels of sedation [3]. In the awake state, they found that patients on PSV had a median of 4 reverse triggered breaths seen in a 25-minute period. While we did review all figures that contained waveforms to determine inclusion/exclusion, we did not focus on systematically analysing the figures pressure support ventilation waveforms for these reasons. We thank the reviewer for their interesting comment, and we have added this as a limitation in our discussion as follows:

*“Furthermore, we excluded spontaneous modes of ventilation in our study. While RT has been described in the literature in assisted modes of ventilation, for instance, secondary to auto-cycling*[3]*, it should be substantially more common in controlled or assist-control modes of ventilation. As such, this is where we directed our search and subsequent analysis.” (see discussion, page 15, line 318 onwards).*

3. Is one figure equal to one waveform? Both terms are used and could be confusing.

All the figures in our systematic search were evaluated to determine if they contained eligible waveforms. Some figures were excluded as they were diagrams/graphs and others because they were from test lungs, animals, or spontaneous modes of ventilation. Following application of exclusion criteria, all included figures were waveforms from controlled or assist controlled modes of ventilation. We have edited the methods section as follows to try to clarify this:

- We have moved the following text from ‘eligibility criteria’ to ‘data collection process’ such that all information regarding what constitutes an eligible waveform is under the same subheading.
  - *“Figures from eligible manuscripts were excluded if there was insufficient information on the figure (single breath recording, isolated waveform of flow or airway pressure), waveforms from animals, test lungs, simulated patients, neonates or pediatric patients, schematic representations, or from spontaneous modes of ventilation (i.e., pressure support, neurally adjusted ventilatory assist, proportional assist ventilation, synchronised intermittent mandatory ventilation).” (Page 7, Line 139 onwards)*
- We have added the following text to clarify the term waveform (see line 149).
  - *“Following application of exclusion criteria, all included figures contained a waveform from a human patient ventilated in assist-control or controlled modes of ventilation. These will be subsequently referred to as “waveforms” for simplicity.” (Page 7, Line 143 onwards)*
- Following this point of the manuscript, we have edited our manuscript at numerous points to use the term waveform more consistently.
- Of note, we continue to use the term ‘figure legend’ as opposed to ‘waveform legend’ as the former is more recognised.

Thank you for this comment, we feel that these changes certainly add clarity to the manuscript, and we hope this satisfies the reviewer.
4. Figure 2: There is a mistake between 197 waveforms/figures included and 181 waveforms/figures excluded.

The reviewer is correct that 181 waveforms is the final number of included waveforms, as opposed to the number that were excluded. We apologise for this error, and we have corrected this in figure 2. The relevant part of figure 2 now reads the following.

*“206 figures excluded for non-invasive ventilation, pure spontaneous ventilation modes (PAV, NAVA, PS, SIMV), schematic representations or incomplete waveforms, waveforms from animals or test lungs. 17 of these excluded figures were removed as they were duplicates from other included studies.”*

We have included the total number of figures excluded (n=206) as well as the number of these figures that were explicitly excluded because they were duplicates (n=17) as we feel this is important information to emphasise.

We have corrected 197 waveforms to 181 waveforms for the final number of waveforms included.
5. Figure 2: The end of the sentence is missing "1737 titles and abstracts excluded for no ……"

We apologise for this error. We have uploaded a new version of figure 2 with the sentence completed reading *“1737 titles and abstracts excluded for no relevance”*
6. L178 and Figure 2: What is "external search"?

The term external search referred to a small number of articles that were identified through review of bibliographies of included in papers. We have updated the manuscript to reflect this:

“External search comprised review of references from studies identified in the initial search” (Page 9 Line 187)

7. L181: "17 figures were excluded […]" from the 181? Why is this not mentioned in figure 2 (and figure 3)?

In figure 2, in the bottom right corner of the figure, in the box regarding exclusions, the final sentence reads “*17 duplicates were identified and removed*”. However, we agree this is important information and should be emphasized. We have therefore re-worded this part of the figure as outlined above in response to a previous comment regarding figure 2 *(see response to minor comment #4).*

8. L140: "We searched Medline and EMBASE for eligible articles" and not Cochrane Central Register of Controlled Trials as mentioned in the abstract?

The reviewer is correct, and we apologise for this oversight. We have edited the methods section under the sub heading “database search” as follows:

*“We searched Medline, EMBASE and the Cochrane Central Register of Controlled Trials” (page 7, line 133)*

This has also been added to figure 2 for clarity.

9. You should mention in the discussion the potential bias related to the difference in the number of figures considered per article. For example, there was 12 waveforms analyzed from Mellot et al. (Heart Lung 2014) versus only one from Kallet et al. (CCM 2006).

Thank you for this important point. We agree that the number of waveforms in a manuscript, but also the focus of that manuscript, could influence the number of waveforms it contains that have evidence of reverse triggering. This is certainly a limitation and has been added to our discussion as follows:

*“Furthermore, the number of eligible waveforms in each study was highly variable, introducing the potential for bias” (Page 14, Line 312)*

As previously stated, estimating the incidence of reverse triggering is unfortunately not possible in the absence of a retrospective database of waveforms.

10. What is "adj3" in the title of Supplementary Figure 1?
Thank you to the reviewer for pointing out this mistake. This was a typographical error and has been removed from the heading of the Supplementary Figure 1. “Adj” refers to adjacent terms and should be explained in the main body of this figure as in the following sentence now in the body of the figure:

*“Numbers of papers in parenthesis; * = truncation term, adj = adjacent terms”*
Best regards

### Reviewer #2:

**General comments:**
I read this manuscript with interest. This systematic review examines the prevalence of undetected reverse triggering (RT) in previously published ventilator waveform figures. The study investigates the presence of RT in ventilator waveforms before and after 2013, shedding light on this previously missed phenomenon. The findings suggest that RT has been present in the literature since at least 1997, with a higher prevalence observed after 2000. While the topic of this study is interesting, there are several concerns.
**Major Comments**
1. The authors assert that prevalence was higher after 2000 due to the use of low tidal volume. However, Table 2 does not provide clear evidence of an association between RT and the use of low tidal volume. It is recommended that the authors clarify this point and propose an analysis to better support this hypothesis. Furthermore, as some of the included studies did not focus on identifying asynchronies, it is unclear how the authors were able to assess that RT is secondary to the management of mechanical ventilation. How were the patients sedated? We need more data on this. This point is crucial to clarify conclusions.

Thank you to the reviewer for this important comment. We agree with the reviewer that the clearest evidence of an association with low tidal volumes would be provided by a comparison of the tidal volumes of cases of reverse triggering versus those without. We carried out a systematic search of the literature and attempted to collate the data that the reviewer is asking for. Unfortunately, many published articles that include waveforms corresponding to ventilator asynchrony are narrative reviews and do not include clinical data at all. Furthermore, the articles that do include such data often did not normalise it to ideal body weight which makes analysis impossible. The association of low tidal volume ventilation with reverse triggering has been suggested by others[1] and we feel that this novel data showing a temporal relationship between the introduction of low-tidal volume ventilation, and an increase in appearance of reverse triggering in the literature is an interesting association. We agree with the reviewer that we cannot definitively state there is an association and we have made the following changes to our conclusion:

We have deleted the following text:

*“Low tidal volume ventilation has previously been suggested as a proposed contributor to reverse triggering and this observation further strengthens that association.”*

And we have replaced it with:

*“The observed temporal relation between the introduction of low tidal volume ventilation and the appearance of reverse triggering is not a definitive association but is of interest given the findings that reverse triggering may be associated with small volume ventilation strategy” (Page 14, Line 294).*

2. It has been described in the literature that RT can be identified with assisted ventilation, why have the authors not included these articles.
Thank you for this interesting comment. Given that reverse triggering occurs after a ventilator delivered breath, it is most observed in controlled modes and assist-control modes. It is possible that reverse triggering could occur in an assisted mode of ventilation, after an auto-cycled or auto-triggered breath, it could be assumed that this should be a rarer event given that a patient in this mode is usually triggering the ventilator. Furthermore, as demonstrated by Longhini et al[3], in the instances that this phenomenon does occur it appears to be relatively infrequent in the awake or lightly sedated patient. While we did review (and subsequently exclude) many pressure support ventilation waveforms, we did not systematically analyse all of the figures for evidence of auto-cycling with reverse triggering as it was felt the yield would be quite low.

We have added this to our discussion regarding limitations as follows:

*“Furthermore, we excluded spontaneous modes of ventilation in our study. While RT has been described in the literature in assisted modes of ventilation, for instance, secondary to auto-cycling*[3]*, it should be substantially more common in controlled or assist-control modes of ventilation” (page 15, line 318)*

3. It may be worthwhile to discuss the potential benefits of monitoring esophageal pressure and/or EAdi in managing asynchronies.
Thank you for this comment, we agree with the reviewer and have added the following paragraph to the discussion:

*“This suggests the potential benefit of monitoring esophageal pressure and/or EAdi in the diagnosis and management of asynchronies or the need for automated methods. Often reverse triggering was mistaken for double triggering and “breath stacking”. There is no direct evidence that monitoring techniques can reduce asynchrony during controlled or assist-control modes of ventilation, but a direct monitor could theoretically improve differentiation between these distinct phenomena, which is the first step towards effective management.” (Page 12, Line 255)*

**Minor comments:**

1. In Table 2: Guiverly needs to be modified by Guervilly.
We apologise for this oversight and thank the reviewer for pointing out this error which we have now corrected.

2. Page 12: There is a mistake in the presentation of the reference: (Leung 1997, Kallet 2002, Kallet 2005)
Thank you for pointing out this error, we have corrected it to be in line with the rest of the references.

### Reviewer #3:

The present systematic review investigates the prevalence of reverse triggering (RT) in the literature containing figures with waveforms. The authors find that, among 66 articles published from 1977 containing 181 waveforms, experts found 21 cases of "possible" (pressure and flow waveform analysis) RT and 25 cases of "definite" RT (additional esophageal pressure or diaphragm EMG). Following 2013, year of the description of RT, the number of undetected cases decreased, but it remains common to miss RT.

The study addresses an interesting research question, which is the underestimation of the prevalence of RT. To address this issue, the authors use an interesting and innovative approach, which is a systematic review based on the retrospective analysis of figures. **Please find my review.**

1) According to the authors, what is the implication of their findings? In what these findings may change our practice. The authors conclude that RT is still underdiagnose and that there is room for education and improvement. However, a systematic review may not be the best way to demonstrate that RT is currently under detected. A prospective cohort study would probably provide a more accurate description. For this reason, I find that the results of this study do not completely support the conclusion.

Thank you to the reviewer for their insightful comments. We view the main implications of this paper as follows: 1) Reverse triggering was present (but misattributed) in the published ventilator asynchrony literature prior to 2013, 2) Reverse triggering continues to be missed after 2013, even in the manuscripts specifically regarding ventilator asynchrony, in the absence of definitive monitoring, and 3) There is a temporal association between the introduction of lung protective ventilation with an increase in reverse triggering in the literature.

We agree with the reviewer that a prospective cohort could give us a more accurate description of the incidence of reverse triggering. Unfortunately, it is very difficult even today to determine the real incidence of reverse triggering in a cohort, because until very recently no algorithm existed to detect it automatically, and very few studies yet have looked at it in a rigorous way recording waveforms over a sufficient period of time and labeling breath by breath all the recordings (unfortunately this is a very time consuming process that we have done a few times in our laboratory). A prospective design may also pose challenges in identifying which cases were reviewed and missed or were just not seen by any clinicians. Finally, given the widespread adoption of low tidal volume ventilation in modern ICUs, it would also not allow us to make inferences regarding this potentially important association.

Given these challenges with a prospective study, it is similarly not possible to accurately determine the true incidence of reverse triggering in a retrospective cohort. Especially in the absence of a cohort of patients with ventilator waveforms. We therefore developed the solution used in the current study to test our broad hypothesis. These are useful to explain whether reverse triggering is really a new phenomenon, if it is related to management, and then nature of its previous misattribution. The fact that reverse triggering is associated with low tidal volumes has been suggested elsewhere [1] and we thought this could be reflected in the "prevalence" over time, knowing that we are most often dealing with narrative review articles (for example, Georgopoulos et al[2]), in which no data are provided regarding tidal volumes or sedation. We did the search in a systematic fashion to ensure we were not missing these important data.

We think that these data present a piece of the puzzle and lend support to arguments from others that low tidal volume ventilation may be contributory to reverse triggering. We therefore believe that our conclusions are modest but valid and agree that the strength of the message must be toned down given the limitations. As such we made the following changes in the discussion:

We have deleted the following text:

*“Low tidal volume ventilation has previously been suggested as a proposed contributor to reverse triggering and this observation further strengthens that association.”*

And we have replaced it with:

*“**The observed temporal relation between the introduction of low tidal volume ventilation and the appearance of reverse triggering is not a definitive association but is of interest given the findings that reverse triggering may be associated with small volume ventilation strategy” (Page 14, Line 294).*

2) There is a lack of consistency between numbers. For instance, it is written "Of a total of 387 figures in these articles, 181 eligible figures with ventilator waveforms were identified for analysis ». And then later "17 figures were excluded as they were reprinted from other included articles. 181 minus 17 makes 164. However, the number or 181 is kept thorough the manuscript.

We apologise for the confusion caused by the description of the articles, figures and waveforms. We have made re-worded this section as follows and hope this provides clarity on the matter:

*“A total of 387 figures were assessed, with 206 figures excluded. Of these excluded figures, 17 were excluded as duplicates from other included articles. In total, 181 eligible waveforms were identified for analysis” (Page 10, Line 207)*

Same questions regarding articles. Of the 66, how many remained after the duplicates were removed?

Removal of duplicate figures did not affect the total number of included articles. The explanation for how we arrived at 66 articles can be seen in figure 2 which is referenced in this part of the manuscript as follows:

“The search extracted 2700 articles and the selection process is described in Figure. 2. After applying our selection criteria, we identified 63 eligible articles for full review, and 2 articles were subsequently identified through an external search that comprised review of references from studies identified in the initial search.”

Finally, it is written that analyses were performed on 197 waveforms. Thorough the manuscript, I suggest being very clear and specific on whether each result is related to the number of articles, figures or waveforms. Otherwise, the reader is lost. This comment also applies to figures. Figure 5 presents RT per waveform. For Figure 4, is it per figure or per wave form.

We thank the reviewer for pointing out the potential for confusion in the description of our analysis.

Regarding 197 waveforms: I believe the reviewer is referring to an error in Figure 2 (search strategy) which we have corrected to read 181 waveforms, which is in line with the rest of the manuscript.

We have attempted to further clarify the description of our analysis by making the following changes:

1. From the methods section onwards we have replaced the term ‘figure’ with ‘waveform’ to be more consistent.
2. Reworded text: “Each waveform constituted our unit of analysis” (Page 8, Line 152)

3) The form of the manuscript needs many improvements. Actually, it was not very pleasant to review a manuscript with two parallel line numbering, without pager numbering and without page break between major sections.

We apologize that the manuscript formatting was unpleasant to read. As per their suggestion we have added page numbering in the revised draft. Unfortunately, the use of page breaks is prohibited in the journal formatting guidelines, and the two parallel line numbering was added not by the authors, but automatically in the generated PDF following upload.

We have also worked to clarify and simplify the overall presentation and avoid redundancy.

4) Although possible, it is quite unusual that none of the authors have disclosures.

The reviewer makes an important comment. The authors have no disclosures that are relevant to this work, but other conflicts are now disclosed.

5) I suggest reducing the length of the introduction to it "catchier". Would the authors consider adding a hypothesis?

Thank you to the reviewer for these comments. We have reworded the introduction such that it is reduced from 704 words to 602 words. We hope the reviewer agrees that it is more concise and to the point.

Additionally, we have reworded the following section to highlight our hypothesis:

*“We wondered whether 1) RT in earlier publications may have gone undetected, with or without attribution to another phenomenon, 2) RT may still be undetected and/or attributed to another type of asynchrony in the absence of direct monitoring of the onset of patient effort. 3) the novelty of this phenomenon may also be related to the era of lung protective ventilation which was generalized mostly after 2000.” (Page 6, 108)*

6) It is written in the introduction that "if the patient's effort is large, this may lead to diaphragm dysfunction [7]". To my knowledge, this is not clearly demonstrated in humans.
The reviewer is correct that this reference pertains to an animal model and as such the sentence has been reworded as follows:

*“I**n an animal model, only when the respiratory effort was large, RT was associated with diaphragm dysfunction” (page 5, line 86)”*

7) Because the criteria use to define RT and hence count the number of missed RTs are very important, I suggest to move them into the main text rather than in the supplement.
Thank you for this comment. As per the reviewer’s suggestion we have moved these criteria from the supplement to the methods section under the sub-heading “Waveform analysis” from line 167 onwards.

8) Search strategy could be added Figure 1
Given the search strategy is relatively long, we feel adding it in its entirety could complicate the figure. However, we agree that the databases searched should be added, and we have included this in Figure 1 as follows:

“*Electronic Database Search: PubMed, EMBASE, and the Cochrane Central Register of Controlled Trials. 4158 Citations Identified.”*

9) "We retrieved 181 eligible figures with waveforms published from 1977 to 2017 with 23 figures (12.7%) published prior to the year 2000". Please provide the number of figures and wave form for each period.

Thank you for this comment. As above we have now made the terminology consistent throughout the manuscript to use the term waveform instead of figure following the description of the methods and initial search. The numbers in this section all pertain to the number of waveforms and it now reads as follows:

*“We retrieved 181 eligible waveforms published from 1977 to 2017 with 23 waveforms (12.7%) published prior to the year 2000 (Fig. 3). 46 waveforms (25.4%) showed evidence of RT, 45 being after 2000 (prevalence of RT 4% vs 28%, X2=4.6 , p=0.03). We classified 21 of these 46 as ‘possible’ RT and 25 as ‘definite’ RT (Fig. 4) Overall, there were 25 of the 46 waveforms with evidence of RT (54%) that were undetected. 17 out of 21 cases of ‘possible’ RT were undetected (80.1%) and 8 out of 25 cases of ‘definite’ RT were undetected (32%).*” (Page 11, Line 217)

10) Table 1 is really too long. By the way, journal titles are sometimes incorrect (ICM, AJRCCM).

Thank you to the reviewer for pointing out these errors in journal titles, which we have corrected in the revised manuscript. Unfortunately given that table 1 is a summary of the articles identified and included in our analysis, there is no way we could shorten it significantly. It could potentially be moved to the supplementary material if the reviewers and editors thought this was appropriate.

11) There are too many illustrations. Actually, data presented in Table 3 and Table 4 could be easily incorporated in the text.
Thank you for the feedback. Before making any changes, we would like to clarify this comment. The reviewer refers to ‘too many illustrations’ but states that tables 3 and 4 are unnecessary. I am not sure if the reviewer is referring to table 3 and 4 as stated or if they actually mean figure 3 and 4. Regarding table 3 – we agree that this could be incorporated into the text. Similarly, table 4 could also be incorporated into the text, but the reviewer states in the subsequent comment (comment 12) that they find this information particularly interesting, so it is not clear if they think the manuscript would be improved by removing this table or not.

If the comment is regarding figure 3 and 4 then we respectively disagree. Previous comments have suggested that the manuscript is confusing with regards to the number of articles, figures and waveforms. We have made an effort to correct these changes and use consistent terminology as per the reviewers’ comments, but we feel that these figures also help in adding clarity to this issue.

12) I found extremely interesting to present the terms used in figure to describe undetected cases of reverse triggering (Table 4). I suggest to highlight a little bit more these findings that have very practical implications for daily practice.

Thank you for the positive feedback. We agree with the reviewer this could be emphasised, and as such we made the following changes in the text:

*“The nature of misattribution of RT to other phenomena is of clinical interest. The majority of cases of misattributed RT in our study were labeled as double triggering or simply ‘breath stacking’. Other cases were labeled as premature cycling or ineffective trigger. It is important for clinicians to be aware of these potential diagnostic errors, as the management can differ significantly, and misinterpretation could lead to an unnecessary increase in sedation in the patient who is asynchronous with the ventilator” (Page 13, Line 267)*

## References

1. Rodriguez PO, Tiribelli N, Fredes S, Gogniat E, Plotnikow G, Fernandez Ceballos I, et al. Prevalence of Reverse Triggering in Early ARDS. Chest. 2021;159:186–95.

2. Georgopoulos D., Prinianakis G., Kondili E. Bedside waveforms interpretation as a tool to identify patient-ventilator asynchronies. Intensive Care Medicine. 2006.

3. Longhini F, Simonte R, Vaschetto R, Navalesi P, Cammarota G. Reverse Triggered Breath during Pressure Support Ventilation and Neurally Adjusted Ventilatory Assist at Increasing Propofol Infusion. J Clin Med. 2023;12:4857.
